# Catalytic Control of Spiroketal Formation in Rubromycin Polyketide Biosynthesis

**DOI:** 10.1002/anie.202109384

**Published:** 2021-11-10

**Authors:** Marina Toplak, Raspudin Saleem‐Batcha, Jörn Piel, Robin Teufel

**Affiliations:** ^1^ Faculty of Biology University of Freiburg Schänzlestrasse 1 79104 Freiburg Germany; ^2^ Institute of Pharmaceutical Sciences University of Freiburg Albertstr. 25 79104 Freiburg Germany; ^3^ Institute of Microbiology Eidgenössische Technische Hochschule (ETH) Zürich 8093 Zürich Switzerland

**Keywords:** antibiotics, collinone, lenticulone, polyketide synthase, redox tailoring

## Abstract

The medically important bacterial aromatic polyketide natural products typically feature a planar, polycyclic core structure. An exception is found for the rubromycins, whose backbones are disrupted by a bisbenzannulated [5,6]‐spiroketal pharmacophore that was recently shown to be assembled by flavin‐dependent enzymes. In particular, a flavoprotein monooxygenase proved critical for the drastic oxidative rearrangement of a pentangular precursor and the installment of an intermediate [6,6]‐spiroketal moiety. Here we provide structural and mechanistic insights into the control of catalysis by this spiroketal synthase, which fulfills several important functions as reductase, monooxygenase, and presumably oxidase. The enzyme hereby tightly controls the redox state of the substrate to counteract shunt product formation, while also steering the cleavage of three carbon‐carbon bonds. Our work illustrates an exceptional strategy for the biosynthesis of stable chroman spiroketals.

## Graphical abstract

Structural snapshots and mechanistic investigation of a key spiroketal synthase provide insights into extensive oxidative backbone rearrangements and spiroketal pharmacophore assembly in rubromycin biosynthesis.

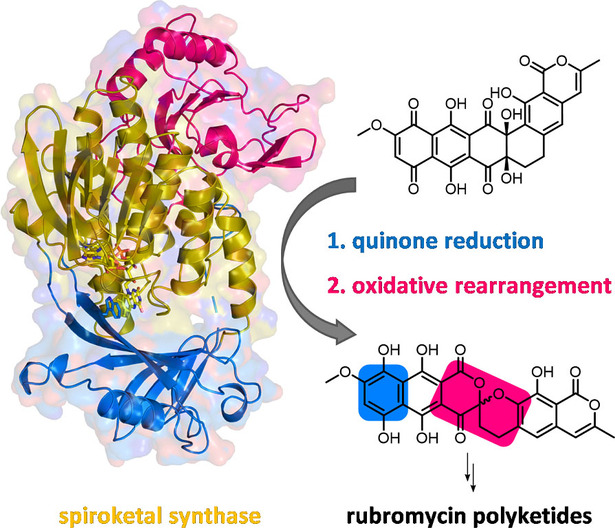

## Introduction

**Figure 1:**
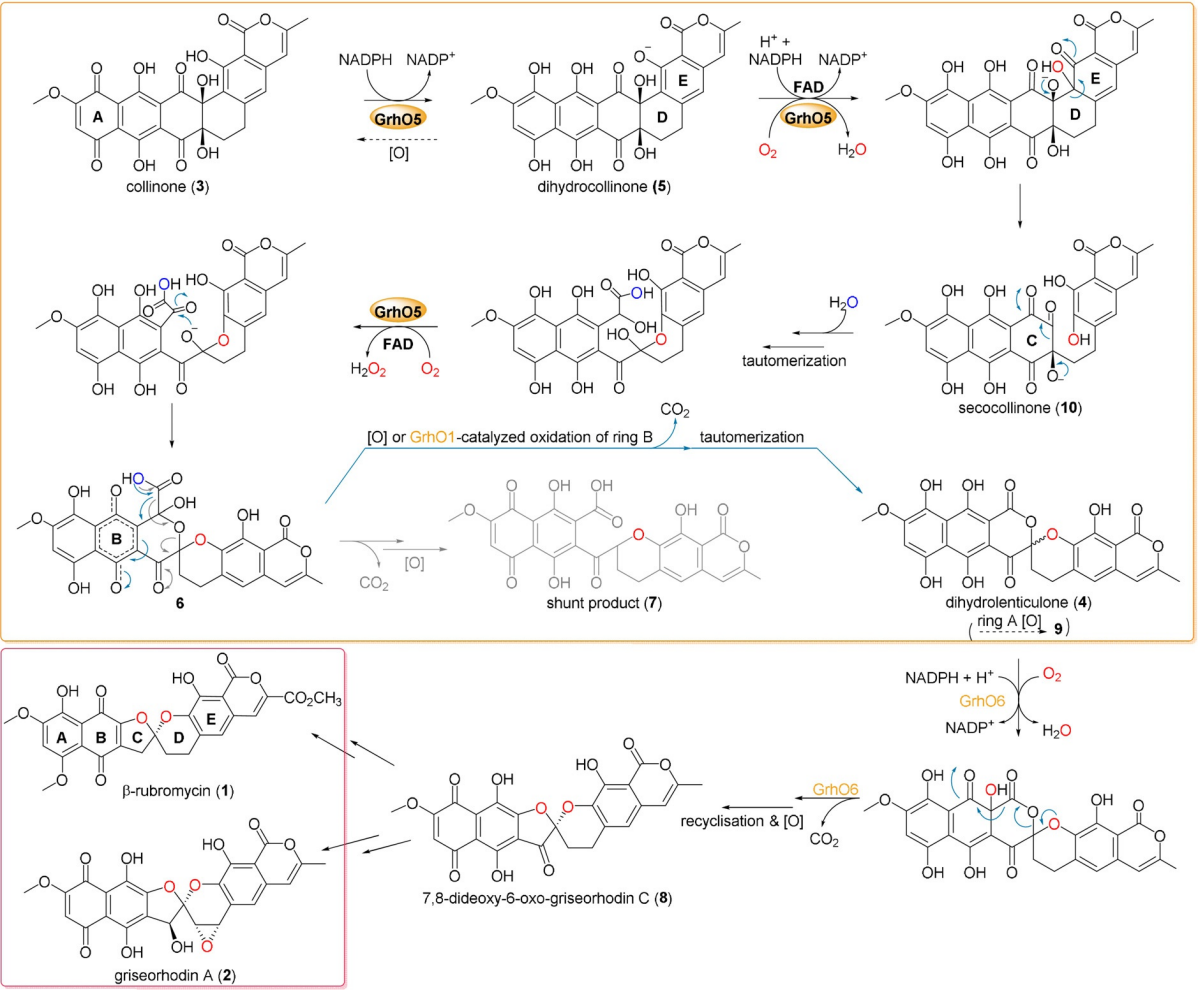
Detailed reaction mechanism proposed for GrhO5 (*orange box*), GrhO1 and GrhO6 leading to the [5,6] spiroketal containing rubromycin polyketides. After the initial reduction of **3** to **5**, GrhO5 is expected to catalyze an aromatic hydroxylation reaction, leading to the formation of unstable secocollinone (**10**, that required methylation prior to structural characterization due to high innate reactivity^[6]^). Compound **10** is further converted into **6**, which may either autooxidize and decarboxylate to **4** (that once more autooxidizes to lenticulone (**9**), analogous to the spontaneous formation of **3** from **5**), or decarboxylate and autooxidize to shunt product **7**. Formation of the shunt product is suppressed by GrhO1, which likely oxidizes ring B of compound **6** to boost **4** formation. Finally, GrhO6 converts **4** into the [5,6]‐spiroketal containing compound 7,8‐dideoxy‐6‐oxo‐griseorhodin C (**8**) that is further processed by pathway‐specific enzymes (not shown) to the mature rubromycins (*red box*). The numbered compounds were previously identified and (partially) structurally characterized (other intermediates are postulated). For details on individual steps, see also main text.

Actinobacteria produce a wide range of bioactive aromatic polyketides, as exemplified by the members of the rubromycin family that feature a characteristic bisbenzannulated [5,6]‐spiroketal pharmacophore,[^1^, ^2^, ^3^] e.g., β‐rubromycin (**1**) and griseorhodin A (**2**). Most rubromycins possess antibiotic or anti‐cancer activities and act as protein inhibitors (e.g., of HIV reverse transcriptase), thus making them possible drug leads.^[4]^ Gene inactivation studies with the **2** biosynthetic gene cluster from *Streptomyces* sp. JP95 implied conventional early steps that generate a polycyclic aromatic backbone, before further tailoring reactions including ring hydroxylations afford the advanced intermediate collinone (**3**) as universal pentangular precursor for the ensuing spiroketal forming steps.[^1^, ^3^, ^5^, ^6^] Recently, two group A flavoprotein monooxygenases (FPMOs) (GrhO5 and GrhO6) and a flavoprotein oxidase (GrhO1) were shown to mediate the drastic remodeling of the **3** backbone and subsequent spiroketal pharmacophore formation.^[6]^ First, GrhO5 generates a [6,6]‐spiroketal moiety as part of the advanced intermediate dihydrolenticulone (**4**), which entails the rupture of three C−C bonds and a CO_2_ elimination step. The NADPH‐ and dioxygen (O_2_)‐dependent GrhO5‐mediated reaction sequence is initiated by an unusual quinone reduction at ring A of **3** yielding dihydrocollinone (**5**). Then, *ortho*‐hydroxylation of phenolic ring E triggers two ring‐cleaving retro‐aldol condensations, carbonyl hydration and several tautomerizations in addition to a putative two‐electron oxidation step that eventually re‐install rings C and D in the form of the [6,6]‐spiroketal (Figure 1).^[6]^ The functional homolog RubL from rubromycin biosynthesis was shown to catalyze the same reaction sequence, pointing toward a universal strategy for spiroketal formation in the rubromycin family. The final product (**6**) proved to be highly unstable and spontaneously formed **4** or the shunt product **7**. GrhO1 presumably mediates hydroquinone oxidation at ring B of **6** and thereby boosts **4** formation while counteracting **7** formation. Finally, GrhO6 hydroxylates phenolic ring B of **4**, which facilitates the decarboxylative ring C opening and subsequent contraction to the mature [5,6]‐spiroketal of 7,8‐dideoxy‐6‐oxo‐griseorhodin C (**8**) (Figure 1). The complex reaction sequence mediated by GrhO5 and RubL and their unexpected functionalities warrant a closer inspection of the underlying catalytic mechanism. Herein, the spiroketal synthases were mechanistically and structurally investigated to understand how they orchestrate and control the distinct redox reactions and backbone rearrangements.

## Results and Discussion

### Structural features and phylogeny of GrhO5 and RubL

**Figure 2:**
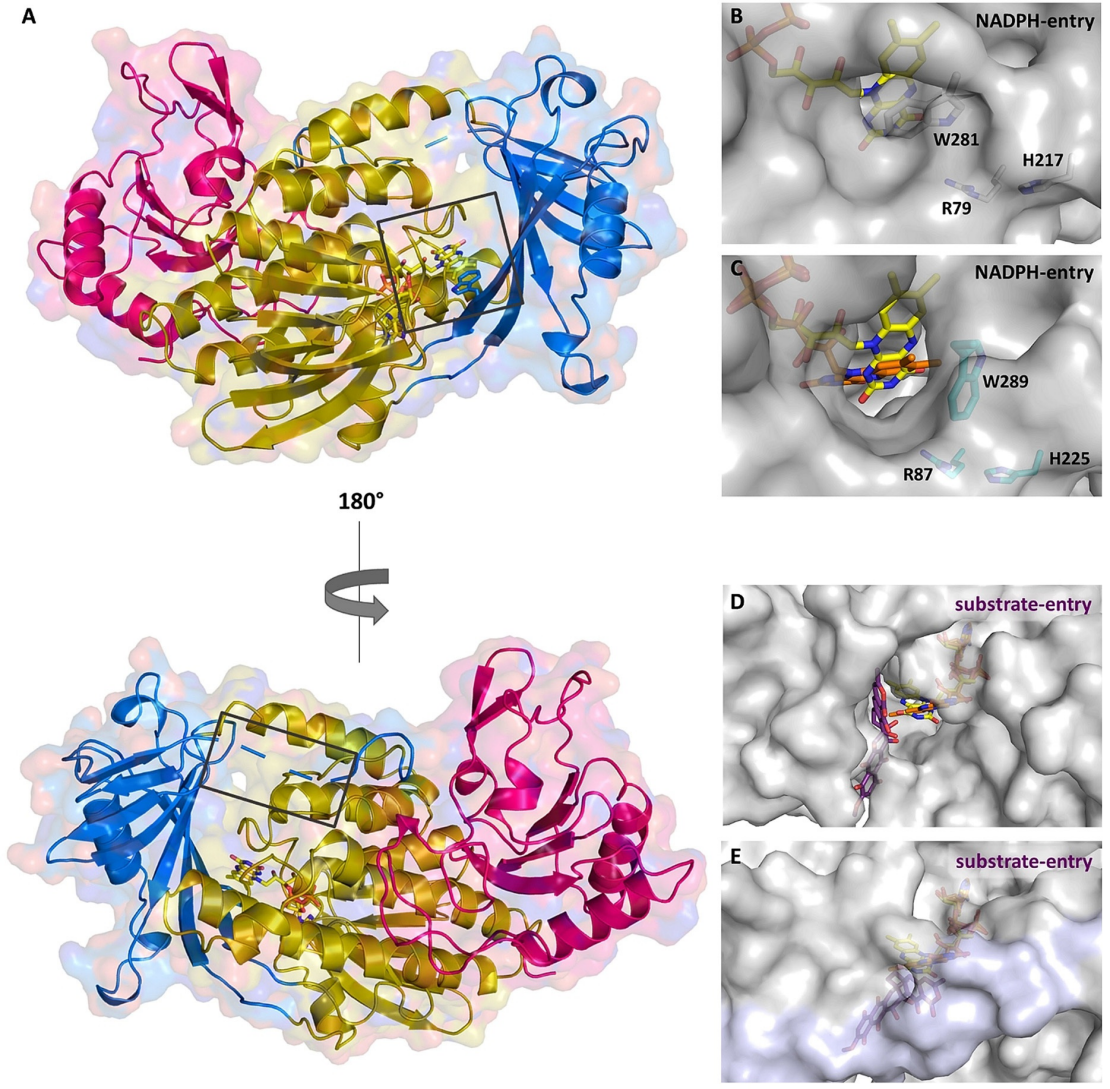
Overall structure of GrhO5 (**A**) and close‐up view of the NADPH‐ (**B**, **C**) and the substrate‐entry sites (**D**, **E**) in GrhO5 and RubL, respectively. **A**, Domain I (*gold*; residues 1–77, 108–188, and 287–386), domain II (*blue*; residues 78–107 and 189–286), and domain III (*pink*; residues 387–537). **B/C**, The NADPH‐entry site. This entry site is blocked by the W281 side chain (W289 for RubL) in ligand‐free (acceptor state; **B**) and **3/5**‐bound GrhO5 (reducible state). Upon rotation of this particular tryptophan residue, as observed in the crystal structure of RubL (catalytic state, **C**), the NADPH‐entry site opens up, thereby probably promoting the expulsion of NADP^+^. **D/E**, Substrate‐entry site. The substrate‐entry site is located on the opposite side of the enzymes and is open both in ligand‐free (acceptor state) and **3/5**‐bound GrhO5 (reducible state; **D**) due to an unordered loop comprising residues 90–110. However, when the protein adopts the catalytic state (**E**), this mobile loop becomes ordered (highlighted in *lilac*) and closes the substrate entry‐site.

**Figure 3:**
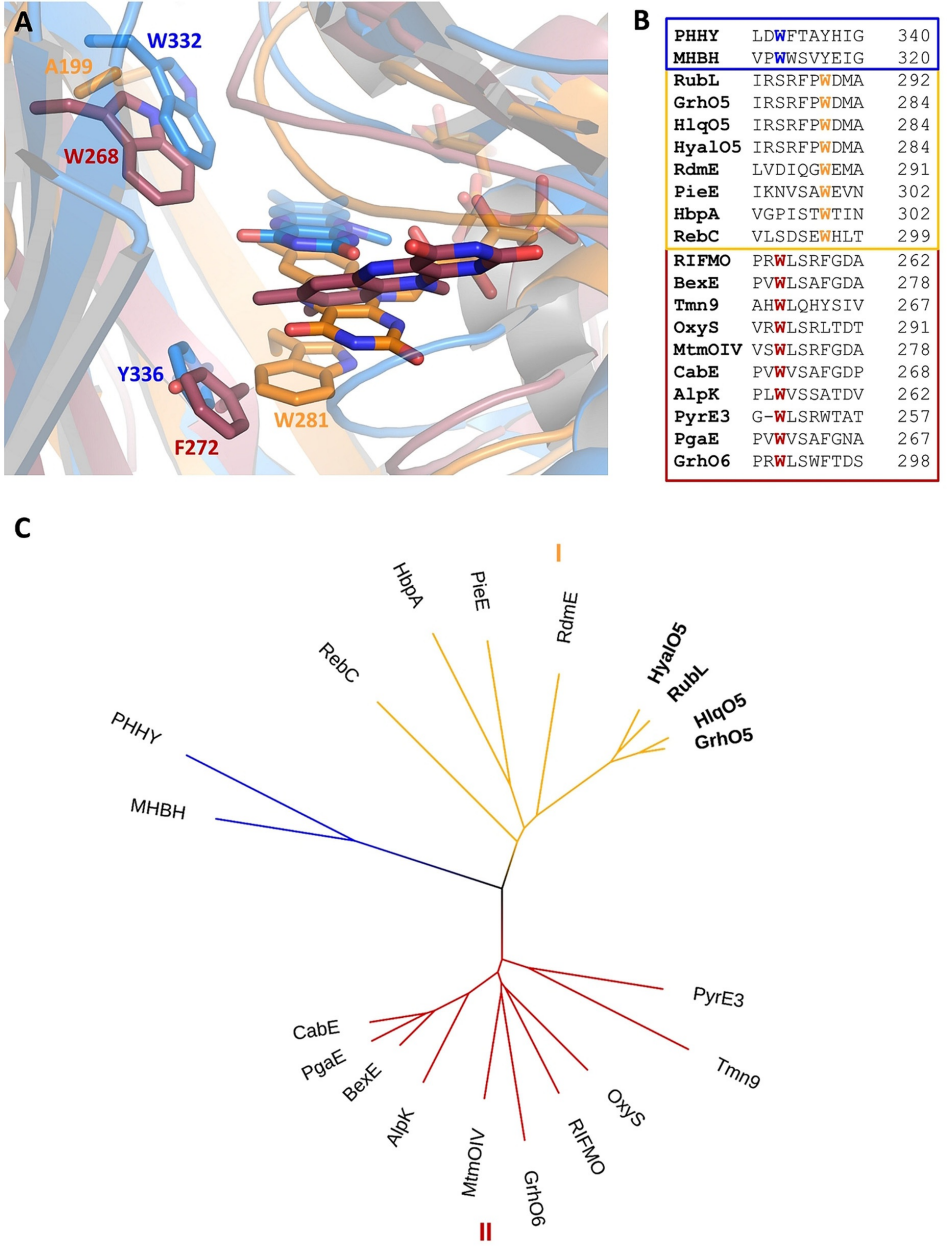
Comparison of group A FPMOs including GrhO5‐like type I (*orange*), MtmOIV‐like type II (*red*), and PHHY‐like (*blue*). **A**, Close‐up views of the active sites with FAD in “OUT” (GrhO5, PHHY) or “IN” (MtmOIV; “OUT” structure not available). **B**, Structure‐based sequence alignment of selected group A FPMOs. **C**, Visualization of the phylogenetic relationships using a bootstrapped distance tree (1000 iterations).^[20]^ See Table S2 for pdb codes and related information and ref. [21] for a comprehensive tree of the entire group A FPMO family.

To investigate the spiroketal synthases, the crystallization of GrhO5 and RubL was attempted under low‐salt conditions in the absence and presence of native substrate **3** and stable shunt product **7**. While no RubL crystals formed under the tested conditions (however, crystals formed under high‐salt conditions, see below), yellow rod‐shaped crystals of native GrhO5 (without **3**/**7**) were obtained after ca. 4–6 months that diffracted up to a resolution of 1.75 Å (for summary of all refinement statistics see Table S1) and enabled structural elucidation by molecular replacement. GrhO5 features three domains similar to other group A FPMOs (Figure 2 A); domains I and II are essential for FAD‐ and substrate binding, respectively, whereas the C‐terminal thioredoxin‐like domain III lacks a catalytically important Cys residue (similar to many other group A FPMOs) that otherwise enables the degradation of H_2_O_2_ in the presence of dithiothreitol.^[7]^ Instead, domain III could be important for protein stability^[8]^ or promote protein‐protein interactions.[^7^, ^9^, ^10^, ^11^] It is noteworthy that the prototype of group A FPMOs, *p*‐hydroxybenzoate hydroxylase (PHBH), is missing domain III entirely. Compared to other well characterized group A FPMOs (Figure 3), high sequence conservation for GrhO5 is only observed in the FAD‐binding domain (Figure 2 A, *gold*; residues 1–77, 108–188, and 287–386) including the GXGXXG (part of the Rossmann fold important for binding of the ADP moiety; GXSXXG for GrhO5/RubL), the GD (contact of Gly300/Gly308 and Asp301/Asp309 with the O3′ of the ribose moiety) and the DG (conserved Asp172/Asp180 and Gly173/Gly181 both involved in FAD and NAD(P)H binding) motives.^[12]^

Group A FPMOs typically feature mobile, non‐covalently bound FAD cofactors to prevent uncoupling (i.e. non‐productive NADPH oxidation and formation of H_2_O_2_ from collapse of the FAD_C4aOO(H)_ oxygenating species) in the absence of the substrates.^[13]^ To achieve that, the FAD cofactors are prevented from reacting with NAD(P)H in the resting state. Although different strategies are known,^[14]^ this typically involves a transition between two conformations of the FAD cofactor—OUT (isolated from the substrate) and IN (close to the substrate). In this work, the combined crystal structures of GrhO5 and RubL suggest three interconvertible catalytically relevant conformers, hereafter referred to as the “acceptor state” (FAD_ox_ in OUT; accepts substrate, no reaction with NADPH), “reducible state” (FAD_ox_ in OUT with bound substrate; reducible by NADPH) and the “catalytic state” (FAD_red_ in IN with bound substrate; reacts with O_2_ for hydroxylation). Moreover, these spiroketal synthases feature two distinct openings to the protein interior and the active site, denoted by “substrate entry site” and “NADPH entry site” (Figure 2 B–E). The GrhO5 structure obtained in the absence of substrate adopts the acceptor state (=resting state) similar to three closely related group A FPMOs from bacterial secondary metabolism (RdmE, anthracycline biosynthesis,^[15]^ RebC, rebeccamycin biosynthesis,^[16]^ PieE, piericidin A1 biosynthesis^[9]^). Notably, the FAD‐OUT conformation in GrhO5 seems to be stabilized by a tryptophan residue (W281) that is properly positioned at the *re*‐side to engage in aromatic π‐π interactions with the isoalloxazine ring. An analogous tryptophan residue was previously reported for some members of group A FPMOs, that is, RdmE,^[15]^ PieE,^[9]^ HbpA (2‐hydroxybiphenyl 3‐monooxygenase)^[10]^ and RebC.[^16^, ^17^] A closer phylogenetic analysis of GrhO5/RubL and related structurally characterized microbial group A FPMOs revealed that enzymes involved in the biosynthesis (e.g., MtmOIV—mithramycin biosynthesis, PieE, RebC, RdmE) or modification (e.g., RIFMO, rifampicin monooxygenase) of natural products are found in two separate clades, henceforth referred to as type I (GrhO5‐like) and type II (MtmOIV‐like). The GrhO5‐type I enzymes including RdmE, PieE and RebC typically function as aromatic hydroxylases and exclusively feature this W fingerprint motif. In contrast, type II members, which include Baeyer–Villiger monooxygenases (e.g., MtmOIV^[18]^) and hydroxylases (e.g., GrhO6^[6]^ or RIFMO^[19]^), as well as distantly related group A FPMOs such as fungal phenol hydroxylase (PHHY) feature a tryptophan residue at a different position perpendicular to the N5 of the FAD‐cofactor that might control solvent accessibility to the active site and/or be involved in stabilization of FAD in OUT (e.g., MtmOIV–W268, PHHY–W332) (Figure 3AB, Table S2 and Figures S1 and S2).

### Enzyme‐substrate interactions and catalytic properties

**Figure 4:**
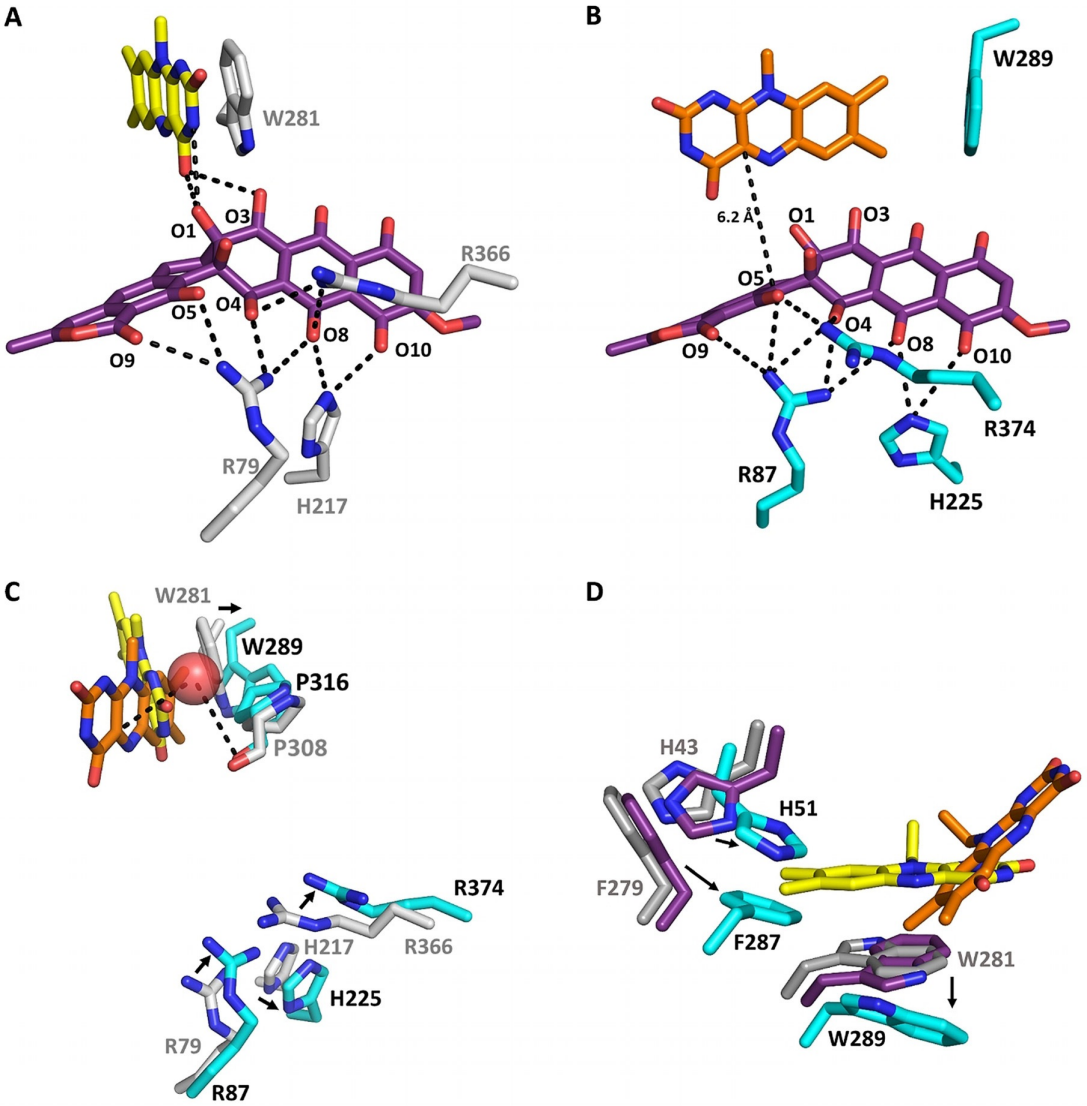
Close‐up view of the substrate binding sites (**A**, **B**), the O_2_ reaction site (**C**) and the FAD surroundings (**D**) of GrhO5/RubL. The active‐sites of GrhO5 (**A**, **C**) and RubL (**B**, **C**) are shown in the “reducible state” (**3**‐soaked GrhO5 with FAD in OUT (*yellow*); **A**, **C**) and the “catalytic state” (RubL with the FAD in IN (*orange*) and **3** modeled into the active site; **B**, **C**), respectively. Panel **C** shows an overlay of **A** and **B** to illustrate different positions of active site residues and the proposed O_2_ reaction site (Cl^−^ binding site, red sphere). Compound **3/5** (*purple*) and selected active‐site residues (*grey* and *teal* for GrhO5 and RubL, respectively) are shown as sticks. Electrostatic interactions (<3.8 Å distance) between several amino acid side chains, the FAD cofactor, and **3/5** are indicated by dashed lines. **D**, FAD surroundings in the acceptor state (*grey*), the reducible state (*purple*) and the catalytic state (*teal*). The movement of FAD to IN is accompanied by changes in the rotameric state of F279 (RubL F287) and H43 (RubL H51) that move toward the center of the active site, resulting in a change of their T‐shaped π‐π‐ to a parallel displaced π‐π stacking interaction in the catalytic state.

To investigate the enzyme mechanism, GrhO5 crystals were soaked with **3**. Gratifyingly, analysis of these crystals revealed clear electron density in the open, solvent‐exposed substrate‐binding site (Figure S3), consistent with the binding of **3** or **5** (possibly formed by X‐ray radiation induced reduction). In this conformation, the O1 and the O3 atoms were positioned in hydrogen‐bonding distance to the C4 carbonyl group and the N3 (only O1) of the isoalloxazine ring of FAD that retained the OUT conformation (Figure 4 and Figure S4). This ternary complex (GrhO5, FAD, **3**/**5**) represents the reducible state, in which the substrate is tightly coordinated by a cluster of basic amino acid side chains comprising H217, R79, and R366 that are in hydrogen‐bonding distance to O8/O10, O4/O5/O8/O9, and O4/O8 of **3**/**5**, respectively (Figure 4 A, Figure S5). Interestingly, while the FAD conformation and active site residues were not affected by **3**/**5** binding, a distinct rotation of the conserved W281 side chain (Figure 3 B and Figure S1) was observed that now adopts an intermediate position (with respect to its final position in the catalytic state, vide infra), while retaining the aromatic sandwich π‐π stacking with FAD (Figure 4 D). This is different from the type I members RebC and PieE, for which substrate soaking of the protein crystals induced the movement of the FAD cofactor to “IN” alongside the rotation of several amino acid side chains.[^9^, ^16^, ^17^]

Many group A FPMO structures feature an FAD cofactor in “IN” even in the absence of substrate, which may in some cases result from the binding of Cl^−^ in the direct vicinity of the isoalloxazine ring that stalls FAD.^[9]^ Similarly, early studies with PHBH suggested that the presence of Br^−^ altered the equilibrium between the two FAD conformations.^[22]^ Hence, crystallization screens under high‐salt concentrations were conducted, in which only RubL crystals formed in presence of shunt product **7** that diffracted to a resolution of 1.6 Å. Indeed, a Cl^−^ was found coordinated between the C4a‐position of the FAD cofactor and the protein backbone including P316 (distance of 3.5–4 Å from the proline carbonyl to Cl^−^; Figure 4 C and Figure S6), which is conserved in the majority of group A FPMOs. Notably, although the active site was partially occupied, clear electron density for the shunt‐product was not observed. However, the FAD cofactor shifted and adopts the “IN”‐conformation, which is stabilized by several hydrogen‐bonds formed between the heteroatoms of the isoalloxazine ring and the protein backbone as well as amino acid side chains. Strikingly, several active site residues have a different rotameric state compared to native and **3**‐soaked GrhO5 with F287 and W289 now stabilizing the FAD conformation by direct T‐shaped (W289 to FAD) and indirect (F287 to W289) π‐π interactions (Figure 4, see Figure S7 for all interactions). At the same time, rotation of these amino acid side‐chains results in the opening of the NADPH‐entry site, which may be beneficial for the efficient release of NADP^+^. Notably, co‐crystallization of RubL with **7** apparently also caused a loop ordering (residues 90–110), which resulted in the closure of the substrate entry site and a more confined reaction chamber (Figure 2DE and Figure S8). Overall, these changes lead to the catalytic state of the enzyme. This is analogous to RebC, in which residues at a similar position (i.e. the domain I‐domain II interface) formed an ordered helix to enclose the bulky substrate.^[16]^ Moreover, other group A FPMOs also possess dynamic structural elements that act as a lid‐like structures for the active site.[^9^, ^14^] It is noteworthy that attempts to co‐crystallize or soak GrhO5/RubL with NADPH were unsuccessful, pointing toward a loose interaction between these constituents, as confirmed by additional experiments outlined below.

### Spectral properties of GrhO5 and RubL

**Figure 5:**
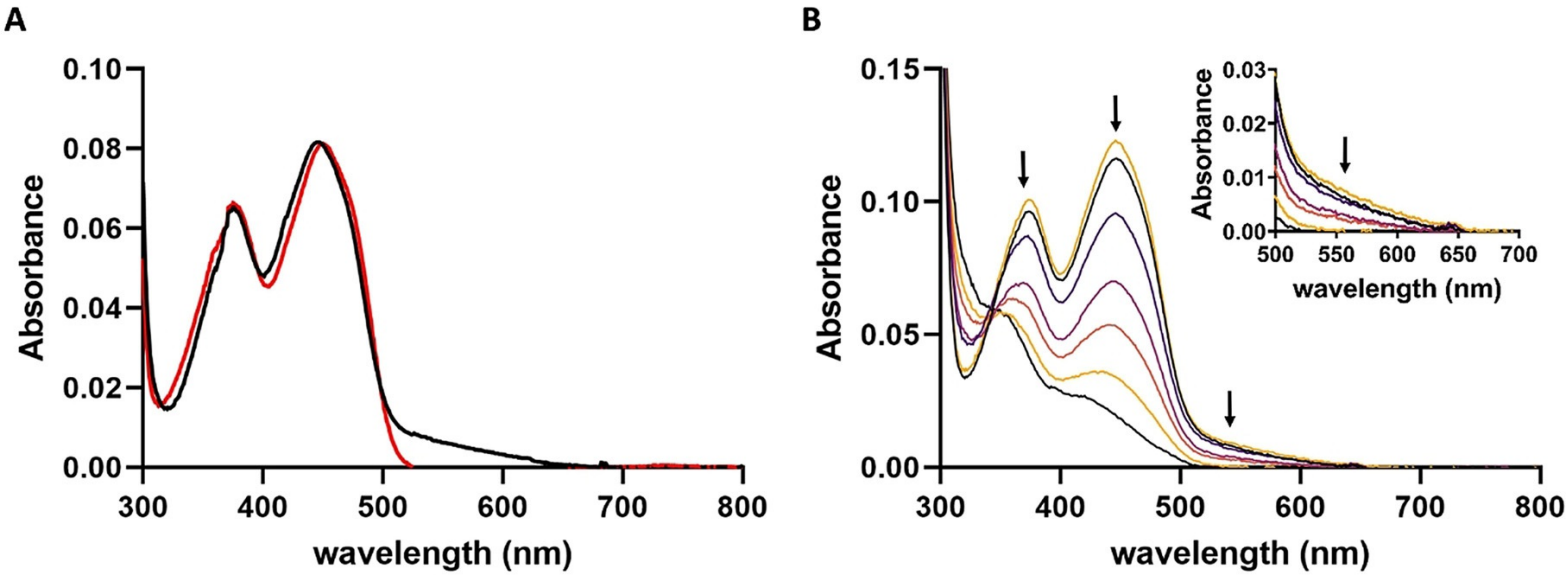
Spectral properties of RubL. **A**, UV‐visible absorption spectra of native (*black line*) and denatured (*red line*) RubL. **B**, Selected absorption spectra recorded in the course of the anaerobic photoreduction of the RubL‐bound FAD cofactor (*top black line*, start spectrum; *top yellow line*, spectrum recorded after reoxidation). The intensities of the peaks at 370 and 450 nm decrease simultaneously, suggesting a direct two‐electron reduction of FAD that goes along with the loss of the CT interaction (shown enlarged in the *Inset*).

To date, interestingly only one member of type I FPMOs (HbpA) has been investigated spectroscopically.^[23]^ Similar to HbpA, analysis of GrhO5 and RubL by UV/Vis spectroscopy revealed a striking long wavelength absorption between 500 and 650 nm, indicative of a charge‐transfer (CT) interaction, in addition to the maxima at ≈370 and ≈450 nm expected for the FAD_ox_ resting state (Figure 5 A). Presumably, the observed CT complex is caused by the aromatic sandwich π‐π stacking of the indole ring of W281 (W289 in RubL) with FAD_ox_.^[24]^ Consistent with that, the long‐wavelength absorption could not be observed after protein denaturation, FAD reduction, or when W289 of RubL was replaced by an alanine (Figure 5 B & Figure S9). Taken together, this provides strong evidence that the “OUT”‐conformation of the FAD observed in the crystal structure also predominates in solution. Most likely, this applies to all members of the new type I that exhibit a similar active site architecture. In contrast, enzymes of type II lack a corresponding aromatic residue (Figure 3) and the UV/Vis spectrum of GrhO6 accordingly does not exhibit such a CT interaction (Figure S10). However, the tryptophan residue of RubL not only seems to be important for the positioning of the FAD cofactor in the OUT‐conformation, but rather for the structural stability and flavin binding in general, as the W289A variant exhibited reduced FAD cofactor loading and higher tendency toward aggregation (Figure S11).

### Reduction potential of RubL‐bound FAD and effect of NADPH

To further investigate FAD dynamics and control of catalysis, the reduction of RubL‐FAD_ox_ by NADPH was first analyzed, which clearly showed that significant NADPH oxidation rates were only observed in the presence of **5**. Hence, in the acceptor state of the spiroketal synthase without substrate, the direct reduction of FAD‐OUT and uncoupling are impeded. To investigate the basis for this behavior, the reduction potential of RubL‐FAD was determined with and without **5** using the xanthine‐xanthine oxidase method.^[25]^ In both cases, simultaneous reduction of RubL‐bound FAD and the reporting dye anthraquinone‐2,6‐disulfonate (*E*°: −184 mV^[26]^) was observed, indicating no significant effect of **5** on the reduction potential, which was calculated at −177±1 mV (Figures S12 and S13). This matches well with previously reported values for group A FPMOs (−163 to −222 mV[^17^, ^27^]). Therefore, **5** binding does not increase the midpoint potential of RubL‐bound FAD and thus is not responsible for the observed acceleration of NADPH oxidation. Hence, instead of being thermodynamically controlled, this kinetic effect may be caused by the gatekeeping W281/W289 side chain that sterically blocks the *re*‐side of FAD in the OUT conformation of RubL to prevent short‐range hydride transfer by NADPH in the acceptor state (as similarly proposed for RebC^[16]^). Importantly, the novel snapshot of the GrhO5 reducible state shows that substrate binding first triggers a distinctive side chain motion of the corresponding W281 that enables the reaction with NADPH, while retaining the sandwich π‐π stacking interaction. Evidently, movement to IN did not occur in the soaked GrhO5 crystals, presumably because FAD unlocking also requires reduction to antiaromatic FAD_red_ in order to disrupt the sandwich π‐π stacking interaction with the flipped W281 side chain (consistent with the previously reported movement of artificially reduced FAD to IN in RebC crystals^[17]^). This mechanistic proposal is supported by experiments with RubL in solution, as the CT complex remained intact in presence of **5**, implying that substrate binding alone is insufficient to trigger FAD movement to IN (Figure S14), which, interestingly, is in contrast to previously characterized HbpA.^[23]^ Importantly, when NADP^+^ (rather than NADPH) was added to the reducible state (RubL‐FAD_ox_‐**5**), a significant loss of the long‐wavelength absorption of the CT complex was observed (which did not occur for the acceptor state, that is, RubL‐FAD_ox_), which likely indicates that the interaction between the W289 side chain and the FAD cofactor becomes weakened by substrate binding. Presumably, this allows the nicotinamide ring to temporarily disrupt the sandwich π‐π interaction by further displacing the flipped W289 side chain and thereby enable hydride transfer to FAD in the reducible state of the enzyme (Figure S14). The now antiaromatic FAD_red_ no longer engages in π‐π stacking interaction; instead conformational changes of the side chains of H51 (H43 for GrhO5), F287 (F279) and also W289 (W281) push the FAD into the IN conformation adopted in the catalytic state and likely promote the departure of NADP^+^ (Figure 4 D).

### Quinone reductase activity, overall substrate turnover and steady state kinetics

To gain a detailed understanding of the quinone reductase functionality, the RubL‐ and NADPH‐dependent conversion of **3** to **5** was characterized. Accordingly, steady state assays were performed under anaerobic conditions by monitoring the decrease of absorption at 650 or 725 nm (for low and high substrate concentrations, respectively), indicative of **3** reduction to **5** (Figure S15). The observed rate constants reached a plateau at ca. 250 to 300 μM **3** in the presence of 1 mM NADPH, resulting in a *k*_cat(app)_ of 4.6±0.2 s^−1^ and a *K*_M(app)_ of 22±4 μM. Notably, when assaying the effect of the NADPH‐concentration on **3**‐reduction activity, RubL showed second‐order kinetics with a *k*_cat(app)_ that linearly increased with the NADPH‐concentration (Figure S16), suggesting a transient interaction with NADPH (group A FPMOs seem to generally interact weakly with NAD(P)H with low *K*_M_ and relatively high *K*_D_ values and promptly release NAD(P)^+^ subsequent to flavin reduction[^28^, ^29^]). Moreover, the titration of NADPH to the RubL‐**3**‐complex did not cause any obvious changes to the FAD spectrum, implying that enzyme‐bound **3** is reduced via direct hydride transfer (without involvement of FAD). Interestingly, additional HPLC analysis suggested that enzyme‐bound **5** is most likely displaced by remaining **3** before hydroxylation can occur, as conversion of **5** into **4** and **7** was only observed subsequent to virtually full reduction of **3** to **5** (Figure S17, WT trace). Next, the overall turnover of **3** into **4** was investigated. As saturation in the reduction reaction is observed at >350 μM **3**, assays were supplemented with 450 μM **3** and 1.5 mM NADPH (previous kinetic assays contained significantly less substrate^[6]^), which, however, resulted in low and fluctuating rates for formation of the final products **4** and **7**. As a decreasing NADPH concentration rapidly affects the turnover number, an NADPH regeneration system was tested. This led to the faster formation of **4** and **7** (*k*_cat_ ca. 0.8–1 min^−1^) and also shifted the reaction equilibrium toward the reduced products and intermediates (**4** and **5** instead of oxidized forms **9** and **3**, respectively), thereby decreasing the number of metabolites in the assay (Figure S18, cf. ref. [6]). In line with previous findings^[6]^ about equal amounts of intermediate **4** and shunt product **7** were obtained (calculated based on the absorption at 500 nm). Overall, these experiments clearly show that **3** reduction by RubL is drastically faster than the ensuing steps under the tested conditions.

### Site directed mutagenesis of active site residues

Based on the ternary crystal structure of GrhO5, several active site residues were identified that participate in **3** binding and presumably catalysis, in particular histidine and arginine residues (GrhO5/RubL: R79/R87, H217/H225, R366/R374). These basic side‐chains may stabilize a deprotonated ring D (distance R79/R87 to phenolic oxygen, ≈3–3.5 Å) and thus increase the nucleophilicity of the phenolate moiety toward the electrophilic FAD_C4aOOH_ oxygenating species. Moreover, a positive electrostatic microenvironment should also favor the subsequent ring‐cleavage and rearrangement reactions, as further discussed below. To study the role of these residues in catalysis and **3** turnover, different active site variants of RubL were generated; R87 and R374 were replaced by a lysine or a methionine (R87K, R374K/R374M) and H225 was substituted by an alanine (H225A). In addition, two active site variants were produced in which E103 (that interacts with R374 in the RubL crystal and may help to position the substrate) was replaced by either a glutamine or an alanine. All variants were obtained in similar yields compared to the wild type protein and seemed properly folded according to size exclusion chromatography (Figure S19 and S20). For all variants, significantly lower **3** reduction rates were observed under anoxic conditions compared to wild type enzyme; however, the most pronounced effect was found for the W289A and the three arginine (R87K, R374K, and R374M) variants with relative activities of ca. 5–6 %, 3–7 %, 7–10 %, and 3–7 %, respectively (Table S3 and Figure S17). In addition, the formation of the final products **4** and **7** was strongly diminished in these variants and could only be observed for the variant with the conservative R374K replacement (Table S4 and Figure S18), suggesting important roles of these residues for aromatic hydroxylation and possibly for the succeeding steps and backbone rearrangements. In contrast, despite their strongly impaired quinone reductase activity, **4** and **7** formation was not affected in the E103A/E103Q or H225A variants, implying that both residues are dispensable for the later steps and could rather be important for substrate binding or positioning.

### Proposed flavin dynamics and control of catalysis

**Figure 6:**
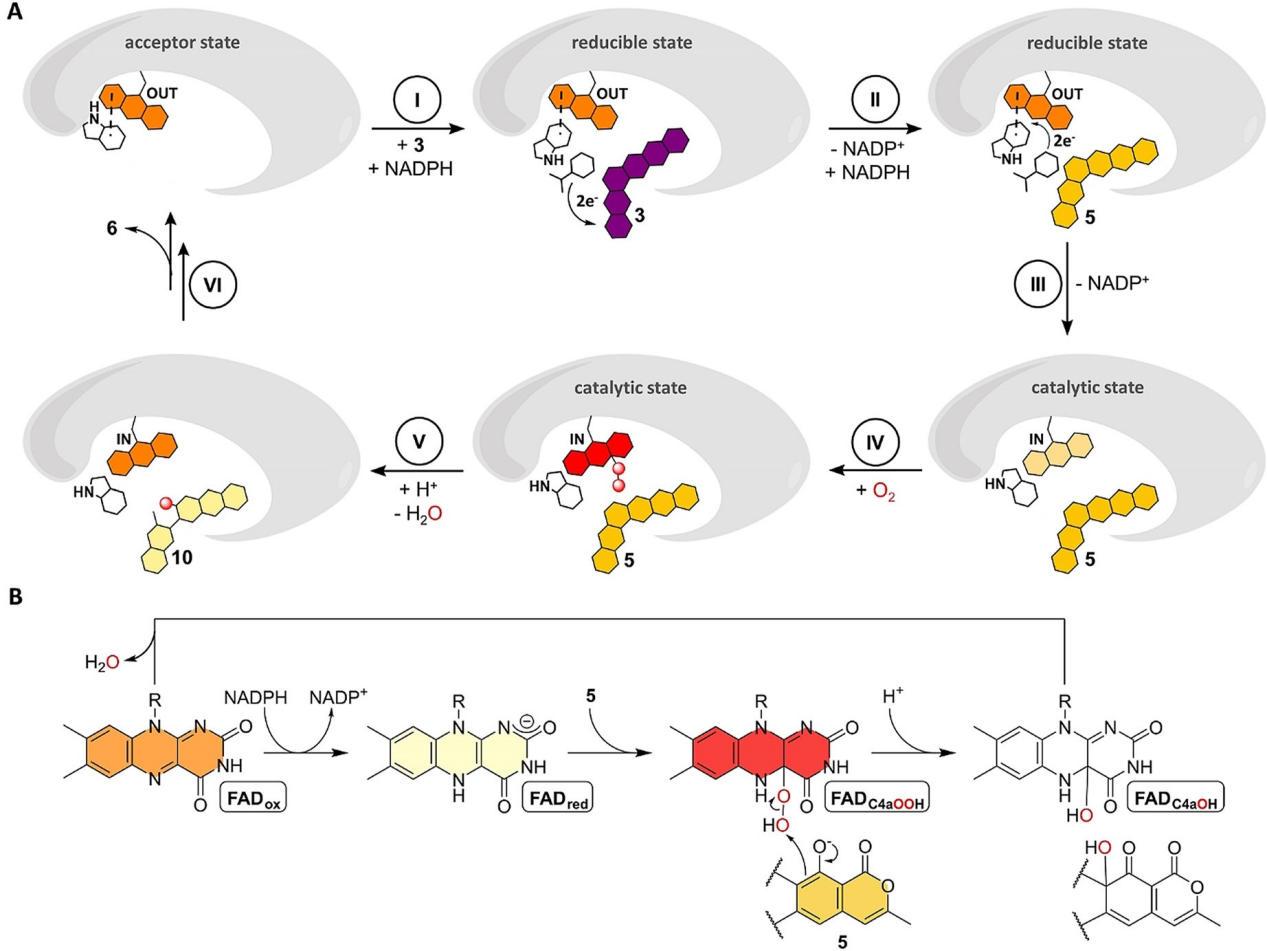
Proposed mechanistic scheme for the conversion of **3** (dark purple) into final product **6** via **5** (yellow) and **10** (light yellow) involving a mobile FAD that adopts the OUT or IN position. The FAD is colored according to its redox state (FAD_ox_, orange; FAD_red_, beige; FAD_C4aOOH_, red). A proposed FAD‐catalyzed two‐electron oxidation during formation of **6** from **10** is not shown (see Figure 1). **A**, Cartoon representation of proposed FAD movements and redox catalysis, see text for details on each step. **B**, Redox states adopted by FAD in the course of the hydroxylation of the phenolate moiety of **5**.

The biochemical and structural characterization portray how catalysis and coupling in the spiroketal synthases are controlled. In the acceptor state, the FAD is locked in the OUT conformation by the gatekeeping π‐π stacked W289 residue (W281 for GrhO5) that has a dual role and furthermore shields the *re*‐side of the cofactor against NADPH. Following the binding of **3**, the reducible state is adopted by flipping of the W289 side chain that exposes FAD_ox_ for reduction, while still impeding cofactor movement via aromatic sandwich π‐π interaction (Figure 4 D). NADPH then transiently displaces W289 by positioning the nicotinamide ring in proximity to FAD_ox_ and **3** (Figure S21 and S22), before the substrate is reduced (probably independent of the FAD cofactor), as the 1,4‐naphthoquinone moiety of **3** is a better electron acceptor than the N5 of the isoalloxazine ring due to a more positive reduction potential (*E*°=−177 mV and 60–80 mV^[30]^ for RubL‐FAD_ox_ and 1,4‐naphthoquinone, respectively) (Figure 6, step I). Following the replacement of NADP^+^ by a new molecule of NADPH, hydride transfer to FAD_ox_ occurs (Figure 6, step II). The now antiaromatic FAD_red_ no longer interacts with W289 and becomes unlocked for the transition to the IN position, presumably driven by movement of the side chains of H43/H51 and F279/F287 as well as W281/W289. This leads to the further **5** sequestration into the active site, coinciding with the ordering of a mobile loop near the lactone moiety of the substrate that closes the substrate entry site and gives rise to the catalytic state of the enzyme (Figure 6, step III). In parallel, the NADPH entry site is fully opened, which may promote the expulsion of NADP^+^ prior to substrate hydroxylation similar to other class A FPMOs.^[31]^

Then, the reaction of FAD_red_‐IN with O_2_ leads to the FAD semiquinone (FAD_SQ_) and superoxide radicals and is likely controlled by a dedicated O_2_ reaction site that facilitates short‐range single electron transfer. The observed Cl^−^ adjacent to the FAD cofactor indicates this site, which is likely optimized to trap the transient superoxide long enough to allow spin inversion and collapse of the radicals to the FAD_C4aOOH_ (Figure 6, step IV).[^28^, ^32^, ^33^] This oxygenating species is then suitably positioned to hydroxylate C4 of **5** (distance of ca. 6 Å between FAD‐C4a and the modelled site of oxidative attack) (Figure 6, step V). The hydroxylated intermediate may then transfer a proton from the C5‐OH group to the formed FAD_C4aOH_ species, which would not only promote the restoration of the FAD_ox_ resting state by water elimination but importantly also lower the activation barrier for the following retro‐aldol ring‐cleavage reaction. The anionic intermediates arising from the deprotonation of C5 and during the backbone rearrangements are likely promoted by the cluster of basic amino acid side chains (R87, R374 and H225 for RubL) surrounding rings C and D of bound **5** in the confined active site (Figure 4). In the course of the [6,6]‐spiroketal forming steps that ultimately lead to **6**, a two‐electron oxidation is required that presumably depends on FAD_ox_ as oxidant. The formed FAD_red_ could then react to the FAD_C4aOOH_ species that subsequently collapses to FAD_ox_ and H_2_O_2_ without a hydroxylation event. Finally, the product **6** is released from the active site concurrent with the restoration of the FAD_ox_ resting state and movement to OUT (Figure 6, step VI). Compound **6** is then taken up by GrhO1/RubI for downstream processing to **4**, before GrhO6/RubN completes [5,6]‐spiroketal biosynthesis with **8** formation.^[6]^

## Conclusion

In this work, we characterized two functional homologs of the key tailoring enzyme involved in the biosynthesis of bacterial rubromycin‐type polyketides, which mediate a drastic oxidative rearrangement of the pentangular precursor **3** to an intermediate [6,6]‐spiroketal moiety en route to the mature natural products. Crystallographic snapshots of binary and ternary enzyme complexes hereby provide a comprehensive picture of FAD dynamics and the control of catalysis to counteract uncoupling. Phylogenetic analyses of group A FPMOs suggested that these spiroketal synthases belong to a newly proposed “type I” subgroup that is characterized by a W fingerprint motif; this residue engages in sandwich π‐π stacking interactions with the FAD cofactor (resulting in a visible CT complex) and adopts a dual role in the resting state of the enzymes by locking FAD_ox_ in OUT and by acting as gatekeeper that shields FAD_ox_ from reduction. This is in contrast to FAD_ox_ of the prototypal PHBH that is generally reducible in OUT but samples between the IN and a possibly exclusive OPEN position to prevent uncoupling in absence of substrate.^[34]^

Notably, an unusual quinone reduction is observed prior to oxygen transfer in these spiroketal synthases. Our data suggests weak interaction of NADPH with RubL and likely a direct hydride transfer to **3** without the involvement of FAD. Quinone reduction appears to be a more common strategy for the activation of pathway intermediates in natural product biosynthesis[^6^, ^35^] but also enables the spiroketal synthases to counteract ring A autooxidation and thereby formation of shunt product **7**.^[6]^ Futile redox cycles, that is, autooxidation and (enzymatic or non‐enzymatic) reduction of quinonic compounds may increase the oxidative stress due to the production of reactive oxygen species, which conceivably contributes to the antibiotic activity and toxicity of rubromycins and makes efficient biosynthetic pipelines and export crucial for their natural producers. For the oxygenation step, FPMOs utilize C4a‐ or N5 oxygenated flavins[^32^, ^36^, ^37^] with group A FPMOs probably exclusively relying on FAD_C4aOO(H)_ species.[^13^, ^14^] For the spiroketal synthases, this is supported by the observed Cl^−^ close to the C4a that likely indicates the O_2_ reaction site in the “catalytic state” (halides act as superoxide analogs^[38]^). A superoxide at the Cl^−^ binding site would approach FAD_SQ_ “face‐on” and thus be ideally positioned to react with C4a and form an anionic FAD_C4aOO_ (Figure 4 C, Figure S6).[^28^, ^39^] Conceivably, the C3‐hydroxyl group of **5** then acts as proton donor for FAD_C4aOOH_ formation to increase the nucleophilicity of the substrate and promote hydroxylation. In this process, the backbone carbonyl group of the highly conserved proline of group A FPMOs (P316 in RubL) may function as an H‐bond acceptor to stabilize and position the FAD_C4aOOH_ intermediate to ensure regiospecific hydroxylation (Figure 4 C). The C4 of modelled **3**/**5** in the active site of the “catalytic state” would then sit at a suitable distance of ca. 6 Å to C4a of FAD for the envisaged hydroxylation (distances from the site of oxidative attack to the C4a are often between 4.5 and 5.5 Å^[40]^).

Interestingly, the subsequent carbon backbone rearrangements may be mostly driven by the high innate energy of the intermediates, which may be a more common principle for complex oxidative backbone rearrangements in polyketide biosynthesis.[^36^, ^40^, ^41^] In support of this, the active site and overall enzymatic features of GrhO5/RubL are not markedly different from group A FPMOs that mediate canonical aromatic hydroxylation reactions, such as RdmE from anthracycline biosynthesis.^[15]^ Consistent with that, no additional shunt products accumulated for the investigated enzyme variants that could point toward possible catalytic roles of individual residues in these rearrangements. Instead, the active site may be particularly important to provide a favorable environment for the spiroketal‐forming steps. Accordingly, an overall positive electrostatic potential through the enrichment of basic amino acid side chains around rings C and D of **5** in the confined active site may promote both the aromatic hydroxylation as well as the spiroketal‐forming carbon backbone rearrangements by lowering the pK_a_ values of the phenolic/alcoholic protons and by stabilizing the postulated anionic intermediates. Overall, this scenario is comparable to the group A FPMO 2‐methyl‐3‐hydroxypyridine‐5‐carboxylate oxygenase, which facilitates ring‐cleavage subsequent to aromatic hydroxylation and also lacks apparent catalytic residues.^[42]^ For GrhO5/RubL, further studies will be required to address the postulated two‐electron oxidation that is likely mediated by FAD_ox_ en route to **6**, which could not be investigated due to the inaccessibility of the corresponding substrate.

Taken together, our study provides new insights into the complex catalysis of a distinctive group A FPMO subtype that features a gatekeeping tryptophan residue for the tethering and shielding of FAD. This work furthermore illustrates an unusual FPMO‐mediated cascade of redox reactions in the form of quinone reduction, aromatic hydroxylation and presumably alcohol oxidation that drives extensive carbon backbone rearrangements and demonstrates how complex chroman spiroketal pharmacophores are assembled in nature.

## Conflict of interest

The authors declare no conflict of interest.

## Supporting information

As a service to our authors and readers, this journal provides supporting information supplied by the authors. Such materials are peer reviewed and may be re‐organized for online delivery, but are not copy‐edited or typeset. Technical support issues arising from supporting information (other than missing files) should be addressed to the authors.

Supporting Information
