# Supplementary material for: Catalytic Control of Spiroketal Formation in Rubromycin Polyketide Biosynthesis
Source: Angew Chem Int Ed Engl. 2021 Nov 10;60(52):26960–70. doi: 10.1002/anie.202109384 (PMC9299503; doi:10.1002/anie.202109384)
Supplement: Supplementary file 1 — Supporting Information [file ANIE-60-26960-s001.pdf]

Angewandte Chemie
Eine Zeitschrift der Gesellschaft Deutscher Chemiker

Supporting Information

Catalytic Control of Spiroketal Formation in Rubromycin Polyketide Biosynthesis

*Marina Toplak, Raspudin Saleem-Batcha, Jörn Piel, and Robin Teufel**

anie_202109384_sm_miscellaneous_information.pdf

Table of Contents

1. Materials and methods **3**

1.1 Recombinant production and purification of RubL 3

1.2 Cloning and recombinant production of MBP-TEV-GrhO5 4

1.3 Determination of the molar extinction coefficient of GrhO5-/RubL-bound FAD 4

1.4 Anaerobic photoreduction of RubL-bound FAD 4

1.5 Determination of the reduction potential of RubL-bound FAD 4

*1.6 Steady state kinetics – reduction of **3** by RubL under anoxic conditions* 5

1.7 Reduction of 3 by RubL-wild type and -variants under anoxic conditions 5

1.8 NADPH-concentration dependence of collinone reduction 6

1.9 Site-directed mutagenesis 6

1.10 Short-time conversion assays (HPLC-analysis based) 7

1.11 10 min conversion assays (HPLC-analysis based) 7

1.12 Structure-based sequence alignment (group A monooxygenases) 7

1.13 Analytical gelfiltration 8

1.14 HPLC detection 8

1.15 Crystallization - RubL 8

1.16 Crystallization - GrhO5 8

1.17 X-Ray Data Collection, and Structure Determination 9

2. Supplementary Figures and Tables **10**

Table S1: Data collection and refinement statistics. 10

Table S2: Overview of all group A FPMOs and their origin... 11

Figure S1: Uncropped structure-based multiple sequence alignment (cf. Figure 3 main text) 15

Figure S2: Surface representation of PHHY indicating the putative gate-keeping role of Trp336. .. 16

Figure S3: GrhO5 ternary complex structure with FAD and collinone (3). 17

Figure S4: LigPlot showing the interactions of GrhO5 (**3**-soaked)-bound FAD........ 18

Figure S5: LigPlot showing the interactions of GrhO5-bound collinone (**3**) with the active site residues Arg79, His217 and Arg366. 19

Figure S6: Putative O2-reaction site in RubL shown in face-on (A) and side-view (B). 20

Figure S7: LigPlot showing the interactions of RubL-bound FAD (“IN”-conformation) with the amino acid backbones and side-chains in the active site. 21

Figure S8: Overlay of the overall structures of GrhO5 (grey) and RubL (teal) with the FAD-cofactor in “OUT”- (yellow) and “IN”- (orange) conformation, respectively. 22

Figure S9: Comparison of the spectral properties of RubL wild type and the W289A variant. 23

Figure S10: UV-visible absorption spectra of native (black) and denatured (red) GrhO6. 24

Figure S11: FPLC-chromatograms recorded in the course of the gel-filtration of RubL wild type (A) and the W289A-variant (B). 25

Figure S12: Determination of the reduction potential of RubL wild type. 26

Figure S13: Estimation of the upper and lower boundary of the reduction potential of RubL... 27

Figure S14: UV-visible absorption spectra of RubL WT (A) and RubL-W289A (B)... 28

Figure S15: Selected (UV-)visible absorption spectra recorded in the course of **3** reduction. 29

Figure S16: Basic kinetic characterization of the first step of collinone (3) turnover... 30

Figure S17: HPLC-analysis of the conversion of **3** (10.3 min, pink line) into **5** (12.4 min, yellow line) catalyzed by RubL wild type or several active site variants. 31

Table S3: Summary of the relative collinone (**3**) reduction rates... 32

Figure S18: HPLC-analysis of the conversion of **3** via **5** into **4** and **7**... 33

Table S4: Summary of the **3** turnover-efficiency observed for RubL wild type and variants. 34

Figure S19: SDS-PAGE analysis of the different fractions collected in the course of the purification of a selected RubL-variant. 35

Figure S20: Analytical size exclusion chromatography of different RubL-variants. 36

Figure S21: Surface representation of RubL, showing the NADPH-entry to the active site… 37

Figure S22: Surface representation of **3**-soaked GrhO5 with the FAD-cofactor (yellow), **3** (purple) and the amino acid side chains coordinating **3**/**5** (grey) shown as sticks. 38

3. References..39

1. Materials and methods

1.1 Recombinant production and purification of RubL

In order to allow for the recombinant production of RubL, the respective gene was purchased, codon optimized for *Escherichia coli* and subcloned into a pET16b-vector (BioCat), in frame with the nucleotide sequence coding for an N-terminal histidine-tag. In addition, a tobacco etch virus (TEV)-cleavage site was inserted 5’ to the gene sequence, to enable the selective removal of the affinity tag prior to protein crystallization. Upon arrival, the dry plasmid DNA was dissolved in ddH_2O to a final concentration of 100 ng µL^{-1} and transformed into *E. coli* BL21(DE3)-cells (Thermo Fisher Scientific) for subsequent gene expression.

For the recombinant production of the protein, TB-medium containing 100 µg mL^{-1} ampicillin was inoculated with a pre-culture grown in LB-medium (supplemented with the same amount of antibiotic) to an optical density at 600 nm (OD_{600}) of ~0.1. The cultures were incubated at 37 °C and 130 rpm, until OD_{600} of ~0.5-0.6 was reached. Then, the temperature in the incubator was set to 18 °C and protein production was induced with 0.1-0.2 mM IPTG. After overnight incubation at 18 °C and 130 rpm, cells were harvested by centrifugation (4000 g for 15 min).

For protein purification, the resulting pellets were resuspended in 50 mM Tris, 300 mM NaCl, 10 % glycerol, pH 7.4 (binding buffer) and cells were lysed by ultrasonication (3 s pulse, 2 s pause; 5 min total pulse time) with a Branson Digital Sonifier (Branson). Then, the lysate was cleared by centrifugation (18000 g for 40-50 min) and loaded onto two 5 mL Ni-NTA FF crude columns (GE healthcare, Cytiva) pre-equilibrated with binding buffer. Unspecifically bound proteins were removed by extensive washing with wash buffer (50 mM Tris, 300 mM NaCl, 30 mM imidazole, 10 % glycerol, pH 7.4) and RubL was eluted with elution buffer (50 mM Tris, 300 mM NaCl, 500 mM imidazole, 10 % glycerol, pH 7.4). Yellow fractions, containing pure RubL were pooled and buffer was exchanged to binding buffer using desalting columns (Hi-trap desalting, GE healthcare/Cytiva). RubL used in activity assays, was concentrated to 350 µM using centripreps (cut-off 30 kDa, Pall), flash frozen in liquid nitrogen and stored at -80 °C until further use.

RubL used for crystallization, after buffer-exchange was incubated with TEV-protease (10:1, m/m) at 4 °C overnight. After removal of the cleaved His-tag and the TEV-protease by a second round of affinity chromatography, the protein was applied to a Superdex 200 pg (HiLoad 16/600) column, pre-equilibrated with 50 mM Tris, 300 mM NaCl, 10 % glycerol, pH 7.4, for further purification. Fractions containing pure RubL were combined, concentrated (to 15 mg mL^{-1}) and stored at 4 °C until further use.

1.2 Cloning and recombinant production of MBP-TEV-GrhO5

Since the initial MBP-GrhO5 construct generated by Frensch et al. [1] did not contain a protease cleavage site allowing for the removal of the solubility tag (MBP-tag) prior to protein crystallization, *grhO5* was recloned to the vector pETHis$_6$-MBP-TEV (between SspI and XhoI, site). Having confirmed the proper insertion of the gene by automated sequencing, the plasmid was transformed into *E. coli* BL21 (DE3)-cells for gene expression. Protein production and affinity purification (incl. removal of the affinity tag) were carried out as described for RubL above.

Like RubL, GrhO5 used for crystallization was applied to a Superdex 200 pg (10/600 HiLoad) column, in this case pre-equilibrated with 20 mM Tris, 50 mM NaCl, pH 7.4, for further purification. Again, yellow fractions corresponding to pure monomeric GrhO5 were combined, concentrated to 200 µM (11 mg mL^{-1}), flash frozen in liquid N_2 and stored at -80°C until further use.

1.3 Determination of the molar extinction coefficient of GrhO5-/RubL-bound FAD

To allow for the determination of the molar extinction coefficients of the protein-bound FAD cofactors, a UV-visible absorption spectrum of both native and denatured GrhO5/RubL was recorded. Based on the assumption that the spectrum of denatured protein equals the one of free FAD (ε_{450}: 11 300 M^{-1} cm^{-1}), molar extinction coefficients of 10 250 M^{-1} cm^{-1} and 11 300 M^{-1} cm^{-1} were calculated for GrhO5 and RubL, respectively.

1.4 Anaerobic photoreduction of RubL-bound FAD

Photoreduction of RubL-bound FAD was carried out in a UV-1800PC photometer (Shimadzu) placed in an anaerobic chamber. A solution containing 11 µM RubL, 25 mM EDTA and 5 µM methylviologen (all components were rendered anaerobic prior to mixing) was prepared in 50 mM Tris, 300 mM NaCl, 10 % glycerol, pH 7.4 and a UV-visible absorption spectrum was recorded between 300 and 800 nm. Then, the sample was irradiated and changes in the UV-visible absorption characteristics were monitored until full reduction of the protein-bound FAD cofactor was observed (no further spectral changes). Finally, the solution was exposed to air for several minutes and a spectrum of “reoxidized” RubL was recorded.

1.5 Determination of the reduction potential of RubL-bound FAD

The reduction potential of RubL-bound FAD was determined photometrically (UV-1800PC photometer, Shimadzu) in an anaerobic chamber using the xanthine-xanthine oxidase method first reported by Massey[2]. First, two solutions, one containing 35 µM RubL, 500 µM xanthine and 10 µM methylviologen and one containing ~40 µM anthraquinone-2,6-disulfonate were prepared in 50 mM Tris, 300 mM NaCl, pH 7.4. Then, these two solutions were mixed (1:1,

v/v) and a UV-visible absorption (starting) spectrum was recorded between 300 and 800 nm. Finally, catalytic amounts of xanthine oxidase (0.06 U, Roche) were added and changes of the UV-visible absorption characteristics were monitored until full reduction of the protein-bound FAD cofactor and the reporting dye was observed (~30 min). By plotting the log($RubL_{ox}$/$RubL_{red}$) at 357 nm (isosbest of anthraquinone-2,6-disulfonate reduction) as a function of the log(dye_{ox}/dye_{red}) at 343 nm (isosbest of RubL-bound FAD reduction) a Nernst-plot was generated, allowing for the calculation of the reduction potential of RubL from the resulting y-axis intercept [3] (quadruplicate determination).

1.6 Steady state kinetics – reduction of 3 *by RubL under anoxic conditions*

Steady-state parameters for the reduction of collinone by RubL wild type were determined using a UV-1800PC photometer (Shimadzu) placed in an anaerobic tent. All assay components were rendered anaerobic and reaction mixtures containing 1 mM NADPH and 33 nM RubL were prepared in 50 mM Tris, 300 mM NaCl, 10 % glycerol, pH 7.4. Then, varying concentrations of collinone (25-350 µM) were added, and absorption changes at 650 nm (ε_{650} of oxidized collinone in buffer pH 7.4: 3 660 $M^{-1}cm^{-1}$) were monitored for 180 s. By plotting the extracted reduction rates as a function of the respective substrate concentrations and by applying a hyperbolic fit, the enzyme characteristic parameters k_{catapp} and K_{Mapp} were obtained (rates at each substrate concentration were determined at least in triplicate).

1.7 Reduction of 3 *by RubL-wild type and -variants under anoxic conditions*

To assay the effect to several amino acid replacements in the active site of RubL on **3** reduction, steady-state assays were performed under anoxic conditions. Reaction mixtures containing 1 mM NADPH and 350 µM **3** were prepared in 50 mM Tris, 300 mM NaCl, 10 % glycerol, pH 7.4 and incubated at room temperature (ca. 23 °C) for 3 min. Then, RubL-WT or one of the variants was added (WT, 233 nM; R87K, 3.2 µM; E103Q, 360 nM; E103A, 390 nM; H225A, 420 nM; W289A, 4.3 µM; R374K, 2.3 µM; R374M, 1 µM) and absorption changes at 725 nm (ε_{725} of oxidized collinone in buffer pH 7.4: 1 060 $M^{-1}cm^{-1}$) were monitored using a UV-1800PC photometer (Shimadzu) for 180 s. Reduction rates were extracted and normalized to the enzyme concentration to obtain apparent k_{cat} values for all variants (reduction rates were determined at least in triplicate for each variant).

For comparison, reaction rates were also determined in the presence of 50 µM **3**. In this case, reaction mixtures containing 1 mM NADPH and 50 µM **3** were prepared in 50 mM Tris, 300 mM NaCl, 10 % glycerol, pH 7.4 and incubated at room temperature (ca. 23 °C) for 3 min. Then, RubL-WT or one of the variants was added (WT, 233 nM; R87K, 0.8 µM; E103Q, 120 nM; E103A, 130 nM; H225A, 140 nM; W289A, 530 nM; R374K, 2.3 µM; R374M, 1 µM) and absorption changes at 650 nm (ε_{650} of oxidized collinone in buffer pH 7.4: 3 660 M^{-1} cm^{-1}) were monitored using a UV-1800PC photometer (Shimadzu) for 180 s. Reduction rates were again

extracted and normalized to the enzyme concentration to obtain apparent k_{cat} values for all variants (reduction rates were determined at least in triplicate for each variant).

1.8 NADPH-concentration dependence of collinone reduction

To study the effect of the NADPH-concentration on the rate of collinone reduction, again a photometric steady-state assay was performed under anoxic conditions. Reaction mixtures containing 66 nM RubL (for the control no enzyme was used) and varying concentrations of NADPH (0.5-4 mM) were prepared in 50 mM Tris, 300 mM NaCl, 10 % glycerol pH 7.4, before adding collinone (250 µM final concentration) and monitoring the decrease in absorption at 725 nm (ε_{725} of oxidized collinone in buffer pH 7.4: 1 060 $M^{-1}cm^{-1}$) for 180 s. Reduction rates were extracted and plotted as a function the respective NADPH-concentration, revealing a liner correlation between the two parameters (rates at each NADPH-concentration were determined in triplicate).

1.9 Site-directed mutagenesis

In order to investigate the role of several active site residues in catalysis, different RubL-variants were generated using PCR-based mutagenesis. In all cases, the mutations were introduced to the pET16b-RubL wild type expression plasmid using both forward and reverse primers carrying the desired nucleotide replacements.

Table 1: Forward and reverse primers used for site-directed mutagenesis, with the codon triplets carrying the desired nucleotide replacements shown in bold.

Variant	Primer	Primer sequence (5'-3')
**R87K**	fwd.	CATCGTACC**AAA**GCAGGCGTGACC
	rev.	CACGCCTGC**TTT**GGTACGATGGGTG
**E103Q**	fwd.	GTAAAGAA**CAG**CTGGAAGGTGACGATG
	rev.	CACCTTCCAG**CTG**TTCTTTACTAAACAGAAC
**E103A**	fwd.	GTAAAGAA**GCT**CTGGAAGGTGACGATG
	rev.	CACCTTCCAG**AGC**TTCTTTACTAAACAGAAC
**H225A**	fwd.	GCGTTGTG**GCT**GTTAAAAATCCGCAG
	rev.	GGATTTTTAAC**AGC**CACAACGCTATAACG
**W289A**	fwd.	GTCGTTTTCCG**GCA**GATATGGCCGG
	rev.	CGGCCATATC**TGC**CGGAAAACGACTAC
**R374K**	fwd.	CTGGCACTG**AAA**AGCGGCACCGCAAC
	rev.	GTGCCGCT**TTT**CAGTGCCAGCTGCAG
**R374M**	fwd.	CTGGCACTG**ATG**AGCGGCACCGCAAC
	rev.	GTGCCGCT**CAT**CAGTGCCAGCTGCAG

Having confirmed the successful introduction of the nucleotide replacements by automated sequencing, the constructs were transformed into *E. coli* BL21(DE3)-cells and protein production and purification were carried out as described for RubL wild type (see above).

1.10 Short-time conversion assays (HPLC-analysis based)

To study the effect of several amino acid replacements on the efficiency of collinone reduction, apart from the steady-state assay conducted under anoxic conditions, also a short turnover assay under aerobic conditions was performed. Generally, reaction mixtures (100 µL final volume) containing 1.5 mM NADPH and 10 µM RubL(-variant) were prepared in 50 mM Tris, 300 mM NaCl, 10 % glycerol, pH 7.4 and incubated at 30 °C for 2 min. Then, collinone (450 µM final concentration) was added and the samples were incubated at 30 °C and 750 rpm for 30 s. All reactions were stopped and extracted with 150 µL EtOAc:FA (9:1, v/v) and the organic layers were analyzed by HPLC-DAD immediately (min. three assays per variant).

1.11 10 min conversion assays (HPLC-analysis based)

To investigate the role of the key active site residues on the efficiency of collinone conversion/lenticulone formation, a 10 min turnover assay was performed. Reaction mixtures (100 µL final volume) containing 1.5 mM NADPH and 10 µM RubL(-variant), 5 mM $MgCl_2$, 10 mM glucose-6-phosphate (G6P) and 2 U of G6P-dehydrogenase were prepared in 50 mM Tris, 300 mM NaCl, 10 % glycerol, pH 7.4 and pre-incubated at 30°C for 2 min. Subsequently, turnover was started by the addition of collinone (450 µM final concentration) and the samples were incubated at 30 °C and 750 rpm for 10 min. The reactions were stopped and extracted with 150 µL EtOAc:FA (9:1, v/v) and the organic layers were analyzed by HPLC-DAD immediately (min. three assays per variant).

1.12 Structure-based sequence alignment (group A monooxygenases)

Sequences for the alignment were selected based on a blastp of the RubL sequence against the sequences/structures deposited in the protein database (PDB) – duplicates and sequences with no significant similarity to RubL were omitted. Instead, the sequences of the known RubL-homologs GrhO5, HlqO5, and HyalO5 were added. Then, a structure-based alignment was generated using the online program T-coffee[4] and visualized in Jalview[5]. Upon comparison of the sequence alignment with the overlaid 3D-structures, errors in the alignment of phenol hydroxylase and *m*-hydroxybenzoate hydroxylase to the other sequences were observed, requiring manual editing of the alignment. Finally, the alignment was visualized using SeaView[6] and a bootstrapped distance tree (1000 repetitions) was generated in the same program. The rooted tree was exported in Newick format and visualized and color-edited using the online program iTOL[7].

1.13 Analytical gelfiltration

Analytical gelfiltration was carried out to determine the biologically active oligomer of RubL wild type and its variants as well as of GrhO5 in solution. Protein samples were applied to a Superdex 200 10/300 GL column, pre-equilibrated with 20 mM Tris, 50 mM NaCl, pH 7.4 and the molecular weight of the protein eluting in the main peak fraction was estimated based on a previously generated calibration curve.

1.14 HPLC detection

Turnover assays requiring offline detection, were analyzed by HPLC using an Agilent 1100 chromatographic system (Technologies), equipped with a VP NUCLEODUR 100-5 C18ec (250 × 4.6 mm ID, 5 µM, Macherey-Nagel) coupled with a UNIVERSAL RP guard column (4 × 3 mm ID, Macherey-Nagel). The column was pre-equilibrated with ddH_2O + 0.1 % TFA (solution A) and acetonitrile + 0.1 % TFA (solution B) 90:10 (v/v) and samples were analyzed at a flow rate of 1.5 mL min^{-1} using the following gradient: 10 % B (0-1 min), 10-100 % B (1-15 min), 100 % B (15-20 min), 10 % B (20-25 min). Considering the strong absorption of all analyzed compounds in the visible-light region, absorption changes at 254, 350, and 500 nm were selected to be monitored by the diode array detector connected to the chromatographic system.

1.15 Crystallization - RubL

RubL crystals were grown in sitting-drop vapor diffusion set-ups (MRC Maxi Plate – Polystyrene; Jena Biosciences). 250 µM RubL (15 mg mL^{-1} presoaked with 1 mM **7**) in 50 mM Tris, 300 mM NaCl, 10 % glycerol, pH 7.4, was mixed with an equal volume of a complex reservoir solution (20 mM DL-arginine hydrochloride, 20 mM DL-threonine, 20 mM DL-histidine monohydrochloride monohydrate, 20 mM DL-5-hydroxylysine hydrochloride, 20 mM trans-4-hydroxy- L-proline, 100 mM BES/TEA pH 7.5 buffer system, 12.5 % PEG 4000 and 2 0% 1,2,6-hexanetriol from Morpheus® II crystallization screen, Molecular Dimensions, Sheffield, UK) at 22 °C. Clusters of RubL needles appeared within 30 days and matured 60 days at 22 °C. Single crystals were dislodged from the cluster, dipped briefly into a drop of reservoir solution without any cryoprotectant, mounted into MiTeGen MicroMounts/MicroLoops, and instantly flash frozen in liquid nitrogen.

1.16 Crystallization - GrhO5

GrhO5 was crystallized using sitting-drop vapor diffusion (MRC Maxi Plate – Polystyrene; Jena Biosciences). 200 µM (~11 mg mL^{-1}) protein solution in 20 mM Tris, 50 mM NaCl, pH 7.4 was mixed with 20 % PEG 3350, 200 mM di-sodium malonate or with 20 % PEG 3350, 200 mM sodium formate (i.e. a crystallization screen condition from JBScreen PACT++, Jena

Bioscience, Jena, Germany) in a 1:1 ratio (0.7 µL/0.7 µL) and plates were incubated at room temperature (23-24°C). After 4-6 months, intense yellow, rod-shaped crystals appeared, which were dipped briefly into a drop of reservoir solution supplemented with 20 % (v v^{-1}) glycerol for cryoprotection, mounted into MiTeGen MicroMounts/MicroLoops, and flash frozen in liquid nitrogen.

GrhO5 + **3** complex crystals were prepared by washing a dislodged single GrhO5 crystal in reservoir solution (2 times) and by subsequently soaking in reservoir solution supplemented with 4 mM **3** in 10 % DMSO for 3 hours prior to cryoprotection and freezing.

1.17 X-Ray Data Collection and Structure Determination

High-resolution X-ray diffraction datasets were collected at the PXI-X06SA beamline (SLS synchrotron, Villigen, Switzerland) at 12.4 KeV (λ=1.0 Å), using an EIGER 16M X (133 Hz, Dectris) detector. Intensity data were collected (ϕ scans of 0.1° over 180° or 360°) up to a resolution of 1.57 Å for RubL crystal and up to 1.75 Å for GrhO5 crystals. The datasets were processed by XDS[8] in I 2 space group (a = 77.32, b = 57.51, c = 122.38; α = γ = 90°, β = 95.19°) for RubL crystal, $P2_1$ space group (a = 46.07, b = 104.44, c = 58.78; α = γ = 90°, β = 104.89°) for GrhO5 and $P2_1$ space group (a = 45.67, b = 103.75, c = 9; α = γ = 90°, β = 95.19°) for GrhO5 in complex with collinone. The X-ray diffraction reflections were merged and scaled using SCALA[9]. Structure determination of RubL and GrhO5 was performed by molecular-replacement with *PHASER*[10] as implemented in CCP4 suite, using a RubL- or GrhO5-structure model generated by the *FFAS* server (https://ffas.godziklab.org/[11]) or SWISS-model server[12], respectively, based on the crystal structure of *Streptomyces purpurascens* RdmE of rhodomycin biosynthesis (PDB ID: 3ihg) as the search model[13]. The structures were modified manually with Coot[14] and refined with *REFMAC*[15] or PHENIX[16] [10]. For omit map calculations Polder OMIT[17] maps tool was used. Restraints for collinone and 1,2,6-hexanetriol were generated using AM1 QM optimization method in eLBOW[18] as implemented in PHENIX[16]. All crystallographic figures were prepared with PyMOL (The PyMOL Molecular Graphics System, Version 2.2.0 Schrödinger, LLC). The structures have been deposited in the Protein Data Bank with accession codes 7OUC for GrhO5, 7OUD for GrhO5 with collinone and 7OUJ for RubL. The data collection and refinement statistics are given in Table S1.

2. Supplementary Figures and Tables

Table S1: Data collection and refinement statistics.

	GrhO5•FAD	***GrhO5•FAD•3***	***RubL•FAD***
PDB code	7OUC	7OUD	7OUJ
Wavelength (nm)	1.0	1.0	1.0
Resolution range (Å)	44.52 - 1.75 (1.84 - 1.75)	44.19 - 2.3 (2.42 - 2.30)	46.08 - 1.57 (1.66 - 1.57)
Space group	P 1 2_1 1	P 1 2_1 1	I 1 2 1
Unit cell (Å), (°)	46.07 104.44 69.23<br>90 104.44 90	45.67 103.75 69.00,<br>90 104.59 90	77.32 57.51 122.38,<br>90 95.19 90
Total reflections	60464 (8879)	87229 (12544)	168341 (24765)
Unique reflections	62285 (8665)	26914 (3911)	70014 (10481)
Multiplicity	3.2 (2.8)	3.2 (3.2)	2.4 (2.4)
Completeness (%)	97.8 (92.7)	97.4 (97.5)	94.4 (96.9)
Mean I/sigma(I)	5.0 (1.2)	5.8 (2.8)	9.4 (2.1)
R-merge (%)	14.6 (63.5)	25.1 (48.6)	4.6 (54.9)
CC1/2	0.99 (0.76)	0.92 (0.83)	0.99 (0.69)
R-work (%)	19.43	22.50	18.30
R-free (%)	23.27	26.29	20.94
Number of non-hydrogen atoms	4429	4255	4264
Macromolecules	3885	3885	3983
Ligands	53	92	87
Solvent	491	278	194
Protein residues	527	533	541
RMS (bonds)	0.010	0.009	0.012
RMS (angles)	1.594	1.543	1.706
Ramachandran favored (%)	98.09	96.18	97.40
Ramachandran allowed (%)	1.87	3.06	2.60
Ramachandran outliers (%)	0.38	0.76	0.00
Rotamer outliers (%)	2.35	4.44	1.48
Clashscore	4.75	7.02	2.98
Average B-factor ($Å^2$)	25.35	10.11	29.35
Macromolecules ($Å^2$)	24.31	10.06	29.15
Ligands ($Å^2$)	15.17	12.50	26.78
Solvent ($Å^2$)	34.68	9.96	34.59

Table S2: Overview of all group A FPMOs and their origin that are displayed in the phylogenetic tree of Figure 3. A, The active sites feature unique conserved tryptophan residues for GrhO5-like type I enzymes. While the FAD cofactor in GrhO5 is held in the "OUT"-position by sandwich π-π stacking with W281, this particular tryptophan is missing in the type II enzymes such as MtmOIV and other group A FPMOs such as PHHY. These enzymes, however, exhibit another conserved tryptophan residue, which is missing in the type I enzymes. Note that GrhO6 and the functional homologs of GrhO5 (HlqO5 and HyalO5) required for formation of other rubromycin polyketides have not been structurally characterized and that GrhO6 clusters with type II enzymes rather than the GrhO5-like type I. All shown enzymes are involved in the biosynthesis or modification of natural products apart from phenol hydroxylase (PHHY[19]), *meta*-hydroxybenzoate hydroxylase (MHBH[20]) and 2-hydroxybiphenyl 3-monooxygenase (HbpA)[21] from bacterial and fungal primary metabolism. For a comprehensive tree including enzymes from primary metabolism and fungal group A FPMOs, see ref. [22].

Enzyme	PDB-ID	Species	Kingdom of life
**PHHY**	1pn0	*Trichosporon cutaneum*	fungus
**MHBH**	2dkh	*Comamonas testosteroni*	bacterium
**RebC**	2r0c	*Lentzea aerocolonigenes*	bacterium
**PieE**	6u0p	*Streptomyces* sp. SCSIO 3032	bacterium
**HbpA**	4z2t	*Pseudomonas nitroreducens*	bacterium
**RdmE**	3ihg	*Streptomyces purpurascens*	bacterium
**GrhO5**	7ouc	*Streptomyces* JP95	bacterium
**RubL**	7ouj	*Streptomyces collinus*	bacterium
**Tmn9**	6ui5	*Streptomyces* sp. NRRL 11266	bacterium
**PyrE3**	5xgv	*Streptomyces rugosporus*	bacterium
**AlpK**	6j0z	*Streptomyces ambofaciens*	bacterium
**BexE**	4x4j	*Amycolatopsis orientalis* subsp. *vinearia*	bacterium
**CabE**	2qa2	*Streptomyces*	bacterium
**PgaE**	2qa1	*Streptomyces* sp. PGA64	bacterium
**MtmOIV**	4k5r	*Streptomyces argillaceus*	bacterium
**GrhO6**	n.a.	*Streptomyces* JP95	bacterium
**RIFMO**	5vqb	*Streptomyces venezuelae* ATCC 10712	bacterium
**OxyS**	4k2x	*Streptomyces rimosus* subsp. *rimosus* ATCC 10970	bacterium

```
                1                                                  20     25
RubL      ---MTSASEP SG-------- -----PSGSA P--------- -VLIVGGSLV GLSTAVFLAR H-----GVRC   39
Grh05     ---------- -M-------- -----SSSGT R--------- -VLIVGGSLV GLSTAVFLAH H-----DVPV   31
Hlq05     ---------- -M-------- -----SNSGT R--------- -VLIAGGSLV GLSTAVFLAH H-----NVPC   31
Hyal05    ---------- -M-------- -----SSSDT S--------- -VLIVGGSLV GLSTALFLTH H-----GIAC   31
RdmE      ---------- -M-------- -----NDHEV D--------- -VLVVGAGLG GLSTAMFLAR Q-----GVRV   31
PieE      -GSHMTGSEG TA-------- -----PDIRV P--------- -VLIVGGGPA GLTAALALSR Y-----GVPH   41
HbpA      ----MHHHHH HS-------- -----NSAET D--------- -VLIVGAGPA GAMSATLLAS L-----GIRS   38
RebC      MGSSHHHHHH SS---GLVPR GSHMNAPIET D--------- -VLILGGGPV GMALALDLAH R-----QVGH   52
RIFMO     ---------- ---------- GS-------- ------HM-I DVIIAGGGPT GLMLAGELRL H-----GVRT   30
BexE      MGSSHHHHHH SS---GLVPR GSHMDA---- ---------- PVVIAGAGPA GLMLAGELRL A-----GIGV   48
Tmn9      ---------- ---------- ---------- -------MSE PVVVVGAGPA GLMLACELAM R-----DVPA   28
OxyS      MGSSHHHHHH SS---GLVPR GS-------- ------HMRY DVVIAGAGPT GLMLACELRL A-----GART   48
MtmOIV    -----GSHMH NSN------- ---------- --ADDAALTT DVVVVGGGPV GLMLAGELRA G-----GVGA   41
CabE      -MAHHHHHHH RS-------- ----DA---- ---------- SVIVVGAGPA GLMLAGELRL G-----GVDV   38
AlpK      ---------- ---------- MEFYDS---- ---------- DVVVVGAGPT GLMLAGELRL A-----GVSV   31
PyrE3     ---------- ---------- ---------- --------SD TVIIAGGGPV GLMLACELGL A-----GVDT   27
PgaE      --AHHHHHHH RS-------- ----DA---- ---------- AVIVVGAGPA GMMLAGELRL A-----GVEV   37
Grh06     MGSSHHHHHH SQDPNSLVPR GSMPDTKGTT DTIDTPGFDF DVIIVGGGPV GMLLASELRI G-----RAKA   65
PHHY      ---------- ---------- ------MTKY SES-----YC DVLIVGAGPA GLMAARVLSE YVRQKPDLKV   39
MHBH      --MQFHLNGF RPGNPLIAPA SPLAPAHTEA VPS-----QV DVLIVGCGPA GLTLAAQLAA F----PDIRT   59

          40               52
RubL      TLVERHPGTS VHPRAVGYYP RTGELLRQAG VEDAAVREAS GFATHRTRAG VTSLAGEVLF SKEELEGDDD  109
Grh05     TLVERHPGTS IHPRAVGYYP RTAELLATVG VEAPAKAAAS GFEKHRTRAG VESLAGEVLF SKEELEGGDE  101
Hlq05     TLVERHPGTS IHPRAVGYYP RTAELLRTVG VEEAAKAAAA GFEQHKTRAG VVSLAGEVLF SKEELAGGDE  101
Hyal05    TMVERHPGTS IHPRAVGYYP RTGELLRQAG AEEAAKRAAS GFENHRTRAG VESLAGEVLF SREELAGADD  101
RdmE      LVVERRPGLS PYPRAAGQNP RTMELLRIGG VADEVVRAD- --DIRGTQGD FVIRLAESVR GEILRTVSES   98
PieE      LLVNRHHGTA HTPRAHLLNQ RTGEIFRDLG IADRVEAHAT PGHLMANHVF MSTFAGPEV- --ARIGAYGN  108
HbpA      LMINRWRSTS PGPRSHIINQ RTMEILRDIG LEESAKSLAV PKEYMGEHVY ATSLAGEEF- --GRIPAWAS  105
RebC      LVVEQTDGTI THPRVGTIGP RSMELFRRWG VAKQIRTAGW PGDHPLDAAW VTRVGGHEVY RIPLGTADTR  122
RIFMO     VVLEKEPTPN QHSRSRGLHA RSIEVMDQRG LLERFLAHGE QFRVGGFFAG L-AA------ ----------   83
BexE      VVLERLPART GESRGLGFTA RTMEVFDQRG LLRRFGEVQT SDQ-----GH FGGI------ ----------   97
Tmn9      VLVDIHPTQR AEAPAMAINA GTLEMLDQRG LAAGLREGTV TFPE----VR FADL------ ----------   78
OxyS      LVLERLAEPV DFSKALGVHA RTVELLDMRG LGEGFQAEAP KLR-GGNFAS L-GV------ ----------  100
MtmOIV    LVLEKLVEPV GHDRAGALHI RTVETLDLRG LLDRFL-EGT QVAKGLPFAG IFTQ------ ----------   94
CabE      MVLEQLPQRT GESRGLGFTA RTMEVFDQRG ILPAFGPVET STQ-----GH FGGR------ ----------   87
AlpK      VVLDKLAEPI KESRALGFSA RTIEEFAQRG LMDRFGEVGV IPV-----GH FGGV------ ----------   80
PyrE3     VVLERHDAPR EPSRGGAINA TVVELFTQRG IMESLRDDGF EFRM----AH FAHI------ ----------   77
PgaE      VVLERLVERT GESRGLGFTA RTMEVFDQRG ILPRFGEVET STQ-----GH FGGL------ ----------   86
Grh06     VVLEKLTERT PHSKAFGLHA RSLESLDRRG LADRFR-EGA RSWNNGHFAG L-DV------ ----------  117
PHHY      RIIDKRSTKV YNGQADGLQC RTLESLKNLG LADKILSEAN DMSTIALYNP DEN--GHIR- ----RTDRIP  102
MHBH      CIVEQKEGPM ELGQADGIAC RTMEMFEAFE FADSILKEAC WINDVTFWKP DPGQPGRIAR HGRVQDTEDG  129

         110
RubL      LGDL------ -TPSRLLLLP QDRLEPLLRD RAVEL-GADL RFGTEL-VSF AEDPE----G VTAVLD----  162
Grh05     LADL------ -TPSSLLLLP QDRLEPILRA RAEEL-GAEL RFGAEL-RSF TQDAD----G VSAVVR----  154
Hlq05     LTDL------ -TPAKLLLLP QDRLEPILRA RAEKL-GADL RFSTEL-VSF AQDAN----G VTAVVK----  154
Hyal05    LGDV------ -TPARLLLLP QDRLEPVLRA RAEEL-GARL LFGTEL-LSF EEDPE----G VTAVLG----  154
RdmE      FDDMVAATEP CTPAGWAMLS QDKLEPILLA QARKH-GGAI RFGTRL-LSF RQHDDDAGAG VTARLA----  162
PieE      GPDRIGEYRA ASPSGLCNLP QHLLEPLLVE AVQEACVGQL RFGHEF-VSL EQDEH----G VTSRIT----  169
HbpA      HPQAHAEHEL ASPSRYCDLP QLYFEPMVVS EA-ALRGADV RFLTEY-LGH VEDQD----G VTARLL----  165
RebC      ATPEH----- -TPEPDAICP QHWLAPLLAE AV----GERL RTRSRL-DSF EQRDDH---- VRATIT----  173
RIFMO     --EWP-ADLD TAHSYVLAIP QVVTERLLTE HATEL-GAEI RRGCEV-AGL DQDAD----G VTAELA----  140
BexE      --PVDFGLLD GAHQAAKTIP QSATEAVLEA WAGEL-GADI RRGHEL-TGV RDDGD----G VAVTVR----  155
Tmn9      --RLAFEKVQ GPREPTHMVL QSRLEKVLID RAVEL-GVDL RWATRL-TGF EEAAD----G SGVTVT----  136
OxyS      --PLDFSSFD TRHPYALFVP QVRTEELLTG RALEL-GAEL RRGHAV-TAL EQDAD----G VTVSVT----  158
MtmOIV    --GLDFGLVD TRHPYTALVP QSRTEALLAE HAREA-GAEI RRGHEV-TGL RQDAE----A VEVTVA----  152
CabE      --PVDFGVLE GAHYGVKAVP QSTTESVLEE WALGR-GAEL LRGHTV-RAL TDEGD----H VVVEVE----  145
AlpK      --PLDYQVIE GGSYGARGIP QARTEGILGG WAREL-GAEV RRGYEV-TAI EDTGT----S VTVEAA----  138
PyrE3     --PLAPERVP GDRAFSFAVP HAQVERRLEE RARSL-GVRV RRSTEI-TSV RQTPD----G VQVTT-----  134
PgaE      --PIDFGVLE GAWQAAKTVP QSVTETHLEQ WATGL-GADI RRGHEV-LSL TDDGA----G VTVEVR----  144
Grh06     --WVDFSLLD SAHNYALLSE QTRTERLLEE RAEEF-GCTI RRGHEV-TAV RQDED----G AEVDVT----  175
PHHY      DTLPGISR-- YHQVVLHQGR IERRILDSIA EISDTRIKVE RPLIPEKMEI DSSKAEDPEA YPVTMTLRYM  170
MHBH      LSEFPHVILN QARVHDHYLE RMRNS-PSRL EPHYARRV-- -----LDVKV DHGAAD---- YPVTVTLERC  187
```

```
                                                                          163                 180
RubL       ---------- ---------- ---------- -------D-G TGGTRTFRSS YLVACDGPRS TVREALKVPR   194
GrhO5      ---------- ---------- ---------- -------D-A DGGESVVRAD YLVAADGPRS TVREALGIGR   186
HlqO5      ---------- ---------- ---------- -------D-A DGTESTLQAD YLVAADGPRS AVRESLGIRR   186
HyalO5     ---------- ---------- ---------- -------A-A DGTSRTVRSD FLVACDGPDS MVRETLKVPR   186
RdmE       ---------- ---------- ---------- -------G-P DG-EYDLRAG YLVGADGNRS LVRESLGIGR   193
PieE       ---------- ---------- ---------- -------DRR TGRDYTVRSD YLIGADGARS RVLAQLGIAL   202
HbpA       ---------- ---------- ---------- -------DHV SGAEYEVRAK YIIGADGAHS LVAQNAGLPF   198
RebC       ---------- ---------- ---------- -------DLR TGATRAVHAR YLVACDGASS PTRKALGIDA   206
RIFMO      ---------- ---------- ---------- --------DG T----RLRAR YLVGCDGGRS TVRRLLGVDF   168
BexE       ---------- ---------- ---------- --------GP AG-EHVLRAG WLVGCDGGRS AVRKAVGFDF   186
Tmn9       ---------- ---------- ---------- -------LAS DAGEEQLRCR YLVGCDGRES IVRKQAGIDY   169
OxyS       ---------- ---------- ---------- --------GP EG-PYEVECA YLVGCDGGGS TVRKLLGIDF   189
MtmOIV     ---------- ---------- ---------- --------GP SG-PYRVRAR YAVGCDGGRS TVRRLAGIGF   183
CabE       ---------- ---------- ---------- --------GP DG-PRSLTTR YVVGCDGGRS TVRKAAGFDF   176
AlpK       ---------- ---------- ---------- --------GA DGSPLSLRAR YVVGCDGARS SVRKLAGIDF   170
PyrE3      ---------- ---------- ---------- ---------- -GDGEVVEGA YLVGCDGSAS LVREQAGIPF   163
PgaE       ---------- ---------- ---------- --------GP EG-KHTLRAA YLVGCDGGRS SVRKAAGFDF   175
GrhO6      ---------- ---------- ---------- --------GP DG-PYTLRAR YVVGTDGGRS LVRRSAGIAF   206
PHHY       SEDESTPLQF GHKTENGLFR SNLQTQEEED ANYRLPEGKE AGEIETVHCK YVIGCDGGHS WVRRTLGFEM   240
MHBH       D--------- ---------- ---------- -------AAH AGQIETVQAR YVVGCDGARS NVRRAIGRQL   221

           195
RubL       QGRGVL--SR HV-------- SIAFGADLRP VLGDRRYSVV HV----KNPQ VTGILVHDDT LTGGTLIVGY   250
GrhO5      EGRGVL--SR HV-------- SIAFGADFAA VLGERRYSVV HV----QNDR VTGILVHDDT LTEGTLIVGY   242
HlqO5      EGRGVL--SR HV-------- SIAFGADFGA VLGDRRYSVV HV----KNDT VTGILVHDDT LTEGTLIVGY   242
HyalO5     HGRGVL--SR HV-------- SIAFGADLKQ VLGDRRYSVV HV----KNPQ VTGILVHDDT LTGGTLIVGR   242
RdmE       YGHGTL--TH MV-------- GVIFDADLSG IMEPGTTGWY YL----HHPE FKGTFGPTDR PDRHTLFVEY   249
PieE       DGATGI--AR AV-------- TTWFEADLSR YSAHRPALLY --MGAVPGSP PADGRVFVSL RPWTEWLHLT   260
HbpA       EGQMGIGDSG SI-------- NIEFSADLSS LCEHRKGDMY YMFRAGSGIN GVGVAALRMI RPWNKWICVW   260
RebC       PPRHRTQVFR NILFRAPELR SLLGERAALF FFLMLSSSLR FPLRALDGRG LYRLTVGVDD ASKSTMDSFE   276
RIFMO      PGEPTRVETL LADVRI---- DVP------V -----ETLTA VVAEVRKTQL RFGAVPAGDG FFRLIVP--A   221
BexE       PGTAATREMF LADLRGVELE PRMIG-ESLP -----GGM-- ---------- -VMVGPLPGG VTRIIVCERD   237
Tmn9       VGDD------ WVIVRGIVGD VA---INRED -----VAPEQ YGLSYTDNGD QFLGAPLSPD VMRVFSAEFS   225
OxyS       PGQDPHMFAV IADARF--RE ELPHG--EGM -----GPMRP YGVMRHDLRA WFAAFPLEPD VYRATVAFFD   250
MtmOIV     PGTEATVRAL IGYVTTPERE -VPRRWERTP -----DGILV LAFPPEGGLG RVVVIEY-TG ---------H   237
CabE       PGTSASREMF LADIRGCEIT PRPIG-ETVP -----LGM-- ---------- -VMSAPLGDG VDRIIVCERG   227
AlpK       PGTEPAIELR FADVAGVQLR PRFSG-ERVP -----GGM-- ---------- -VMVLPMGPD RCRVIYFDSS   221
PyrE3      PGVDPDFHGL WGDIKVEPGA PV---LERIG -----ARQYE LGLC------ --MVAPIGPD TVRVITGEFD   217
PgaE       PGTAATMEMY LADIKGVELQ PRMIG-ETLP -----GGM-- ---------- -VMVGPLPGG ITRIIVCERG   226
GrhO6      PGTGGRVTAR LADVVLADRE NAPMGMERTE -----RGLL- FCVPLDDTYH RVATFDYEQG ---------K   261
PHHY       IGEQTDYIWG VLDAVPASNF P-DIRSRCAI HSAESGSIMI IPREN-NLVR FYVQLQA--- -------RAE   298
MHBH       VGDSANQAWG VMDVLAVTDF P-DVRYKVAI QS-EQGNVLI IPREGGHLVR FYVEMDK--- --------LD   278

           251                                          285  289
RubL       RPEDGESLED FTDDRCAELV GAAVGAPGVE VTIRSRFPWD MA-------- ----EQVAES FVHGRVLLAG   308
GrhO5      DPEKGEGLDD FTDARCAELV SAAIGSDDVA VTIRSRFPWD MA-------- ----ELVADA FVSDRVLVAG   300
HlqO5      DPEKGESLDD FTDARCAELV SAAIGSDAVE VTIRSRFPWD MA-------- ----ELVSET FVQDRVLIAG   300
HyalO5     DAEHEEGLDT FTDARCAELV SAAVGAPGVE VTIRSRFPWD MA-------- ----EQIAEG YVHGRVLLAG   300
RdmE       DPDEGERPED FTPQRCVELI GLALDAPEVK PELVDIQGWE MA-------- ----ARIAER WREGRVFLAG   307
PieE       FPPPTADVDV EDHEAVRAGI RESIGDPTVD VTIKNVSAWE VN-------- ----SAVAPR YASGRVFCVG   318
HbpA       GYEKSKGTPE ITKEEAKKII HEIIGTDEIP VEVGPISTWT IN-------- ----QQYAVR NTSGRVFCMG   318
RebC       LVRRAVAFDT ---------- ---------E IEVLSDSEWH LT-------- ----HRVADS FSAGRVFLTG   315
RIFMO      QGLSA-DRAA PTLDELKRCL HATAGTDFGV HSPRWLSRFG DA-------- ----TRLAER YRTGRVLLAG   278
BexE       APPRR-RTGP PPFHEVADAW KRITGIDISA AEPVWLSAFG DA-------- ----TRQVTE YRRGRVLLAG   294
Tmn9       TEPPEFEDGP ATLEQLGDAV KRLTGKELKA TEAHWLQHYS IV-------- ----TRNAEQ YRKGRVFIAG   283
OxyS       RPYAD-RRAP VTEEDVRAAL TEVAGSDFGM HDVRWLSRLT DT-------- ----SRQAER YRDGRVLLAG   307
MtmOIV     SPAAD-EGPV TLEDLGAAVA RVRGTPLTLT EPVSWLSRFG DA-------- ----SRQAKR YRSGRVLLAG   294
CabE       APARR-RTGP PPYQEVAAAW QRLTGQDISH GEPVWVSAFG DP-------- ----ARQVSA YRRGRVLLAG   284
AlpK       QPLRT-APEA ITFEEVADSW QRLTGEDISG ATPLWVSSAT DV-------- ----SRQAAQ YRKGRVFLAG   278
PyrE3      VPSPP-ADQE VGFDELRAAV ARIAGVELDG VPG-WLSRWT AT-------- ----SRQAER YREGRILLAG   273
PgaE       TPPQR-RETP PSWHEVADAW KRLTGDDIAH AEPVWVSAFG NA-------- ----TRQVTE YRRGRVILAG   283
GrhO6      EAGSE-LGFE EFKDSVRAI- ---WGDDMGA SEPRWLSWFT DS-------- ----ACQAET YRAGRILLAG   314
PHHY       KGGRVDRTKF TPEVVIANAK KIFHPYTFDV QQLDWFTAYH IGQR------ ----VTEKF- SKDERVFIAG   357
MHBH       ADERVASRNI TVEQLIATAQ RVLHPYKLEV KNVPWWSVYE IGQRICAKYD DVVDAVATPD SPLPRVFIAG   348
```

```
309
RubL      DAAHVVPPTG GYGANTGIAD AHNLAWKLAL VAAGVAGPGL VETYDAERRP VAVYTAEQGS LQ--------   370
Grh05     DAAHQIPPTG GYGANTGIAD AFNLSWKLAH VLAGTAGRAL LDTYDEERRP VGLYTARQGS LQ--------   362
Hlq05     DAAHQIPPTG GYGANTGIAD AFNLAWKLAL VLAGTAGSAL LDSYQEERGP VGTYTAQQGS LQ--------   362
Hyal05    DAAHVIPPTG GYGANTGIAD AHNLAWKLAL VLQGSAGTAL LDTYDAERRP VGEYTAQQAS LQ--------   362
RdmE      DAAKVTPPTG GMSGNAAVAD GFDLAWKLAA VLQGQAGAGL LDTYEDERKV AAELVVAEAL AI--------   369
PieE      DAVHQNPPTN GLGLNSAVAD SFNLCWKLKL ALEGLAGPGL LDTYHDERQP VGRQIVDRAF RSMVDLIGIP   388
HbpA      DAVHRHTPMG GLGLNTSVQD AYNLAWKLAL VLKGTAAPTL LDSYDAERSP VAKQIVERAF KSLSTFPPVF   388
RebC      DAAHTLSPSG GFGMNTGIGS AADLGWKLAA TLRGWAGPGL LATYEEERRP VAITSLEEAN VNL-------   378
RIFMO     DAAHIHPPTG GQGLNLGIQD AFNLGWKLAA AIGGWAPPDL LDSYHDERHP VAAEVLDNTR AQMTLLSLD-   347
BexE      DAAHVHLPAG GQGMNAGIQD AVNLGWKLAA VVRGTARADL LDTYHGERHP VGVRLLMNTR AQ-GLLFLNG   363
Tmn9      DAAHVHYPYN GQGLGTAIGD AVNLGWKIAA EVHGWAPADL LDSYHVERHL AGRLACMNIQ AQLALLY-PR   352
OxyS      DACHIHLPAG GQGLNLGFQD AVNLGWKLGA TIAGTAPPEL LDTYEAERRP IAAGVLRNTR AQAVLIDPD-   376
MtmOIV    DAAHVHFPIG GQGLNTGLQD AVNLGWKLAA RVRGWGSEEL LDTYHDERHP VAERVLLNTR AQLALMRPDE   364
CabE      DSAHVHLPAG GQGMNVSVQD SVNLGWKLAA VVSGRAPAGL LDTYHEERHP VGRRLLMNTQ AQ-GMLFLSG   353
AlpK      DAAHIHLPIG AQGMSAGVQD AVNLGWKLAL DISGRAPQGL LDTYHSERHP VGQRILTNTL AQ-RILYLGG   347
PyrE3     DAAHTLFPLG GQALGTGIED AVNLGWKLAA TVQGWAPPSL LDSYHEERHA AGARACASTR AQTTIMR-SL   342
PgaE      DSAHIHLPAG GQGMNTSIQD AVNLGWKLGA VVNGTATEEL LDSYHSERHA VGKRLLMNTQ AQ-GLLFLSG   352
Grh06     DAAHTHFPVG GQGVNLGLQD ALNLGWKLCA DINGWAGEGL LDTYDAERQE PARQVLANTR AQIALMNPD-   383
PHHY      DACHTHSPKA GQGMNTSMMD TYNLGWKLGL VLTGRAKRDI LKTYEEERQP FAQALIDFDH QFSRLFSGRP   427
MHBH      DACHTHSPKA GQGMNFSMQD SFNLGWKLAA VLRKQCAPEL LHTYSSERQV VAQQLIDFDR EWAKMFSDPA   418

371
RubL      --LALRSGTA TPEQQAAVA- ---------- ---------- --------DA VTVTSGQAYR STAVVGEPDG   409
Grh05     --LAVRSRTA TEEQREAAH- ---------- ---------- --------DA MRVTMGQAYP SGAFVADAGA   401
Hlq05     --LALRSGTA TEEQRAATD- ---------- ---------- --------DA MTVTMGQAYR SGAFIPEAGA   401
Hyal05    --LAVRSGTA TPEQRAELV- ---------- ---------- --------DE LTVTMGQSYR STAIVPEAGH   401
RdmE      --YAQRMAPH MAEVWDKSV- ---------- ---------- --------GY PETLLGFRYR SSAVL--ATD   406
PieE      QALGFTEGQS PEEQWRLLDT LHEDTEEARQ RRAAL-AAAT -AAIHGQANA HGVELGYRYR TGALVPDGTP   456
HbpA      EALSLPPAPT ESEMAEALVR LKDASEEGAK RRAALRKAMD -ATIIGLGGG HGVELNQRYV SRAVFPDGTP   457
RebC      ---------R RTMDRELPPG LHDDGPRGER IRAAVAEKLE RSGARREFDA PGIHFGHTYR SSIVCGEPET   439
RIFMO     PGPRAVRRLM AE-------- ---------- ---------- ---LVEF-PD VNRHLIEKIT AIAVRYDL--   383
BexE      AEMQPLRDVL AE-------- ---------- ---------- ---LTG-YPD VARHLAAMVS GLEIAYDV--   399
Tmn9      PLARYMREMM GE-------- ---------- ---------- ---FLK-FDE VNVFLAEIVT NLGPAVPIAY   390
OxyS      PRYEGLRELM IE-------- ---------- ---------- ---LLHV-PE TNRYLAGLIS ALDVRYPM--   412
MtmOIV    QHTTPLRGFV EE-------- ---------- ---------- ---LLGT-DE VNRYFTGMIT GTDVRYATF-   401
CabE      DEMQPLRDVL SE-------- ---------- ---------- ---LIR-YDE VSRHLAGMVS GLDIRYEV--   389
AlpK      DEITPMREVL AE-------- ---------- ---------- ---LMGSHVS VQRHLAGMVT GLDIRHDV--   384
PyrE3     ARVGELRALL TE-------- ---------- ---------- ---LAG-LEE VNAYLVRMVG GID-------   373
PgaE      PEVQPLRDVL TE-------- ---------- ---------- ---LIQ-YGE VARHLAGMVS GLEITYDV--   388
Grh06     PYVTQLRELF QD-------- ---------- ---------- ---LMRK-DQ VNHHIAEMLS GVRVRYDLP-   420
PHHY      AKDVADEMGV SMDVFKEAFV KGNEFASGTA INYDENLVTD KKSSKQEL-A KNCVVGTRFK SQPVVRHSEG   496
MHBH      KE--GGQGGV DPKEFQKYFE QHGRFTAGVG THYAPSLLTG -QAKHQAL-A SGFTVGMRFH SAPVVRVCDA   484

410
RubL      ADLPVASDPR ELRGA--PGT RAPYVELLRG GETVSTLDLF GRD-FVLLTG EHGREWISAA VSASAGLGLK   476
Grh05     DPLPLTSDPR TLRGE--PGT RAPYVVLERD GAPLSTLDLF GDG-FVLLVG ADGGSWAEAA GEAAAGLGVG   468
Hlq05     QRLPLTSDPR TLRGE--PGT RAPHVVVERD GTPVTTLDLF GSG-FVLLAG ADGGSWAAAA AAAGSALGTD   468
Hyal05    EQCPDPCDPR TLRGA--PGT RAPYVILEGR GGPVSTLDLF GEG-FVLLAG ERGTPWAVAA TAGARRWDAD   468
RdmE      DDPARVENPL TPSGR--PGF RGPHVLVSRH GERLSTVDLF GDG-WTLLAG ELGADWVAAA EAVSAELGVP   473
PieE      EPADERDPEL YYRATTWPGA RLPHAWLENG RHRCSTLDVT GRGRFTLLTG PGGEPWRDAA RDAALDTGVE   526
HbpA      DPGFVRDQEF FYQASTRPGA HLPHVWLTEN QRRISTLDLC GKGRFTLLTG LSGAAWKHEA EQVSQSLGIE   527
RebC      EVATGG---- -WRPSARPGA RAPHAWLT-- -PTTSTLDLF GRGFVLLSFG TTDGVEAVTR AFADRHVPLE   501
RIFMO     ------GDGH DLVGRRLRDI P----LT--- -EGR-LYERM RGGRGLLL-- --DRTGRLSV S------GWS   428
BexE      -----GGGSH PWLGRRLPRL E----LDR-G GRPSSTAELL RPARGLLLDF AGNAALRDRA A------PWA   453
Tmn9      EGVPEPVEGD RLLGRRLPKV Q----IKTAD GD-MGVAETL QSGRGVLLDL SGDASAQEES -------GWA   448
OxyS      ------AGEH PLLGRRVPDL P----LVTED GTRQ-LSTYF HAARGVLLTL GCDQPLADEA A------AWK   465
MtmOIV    -APAAPARPH PWAGRFAGGL V----LSGPS GEPVPVAELL RSARPLLLDL AGRADLRE-- ----ATRPWS   460
CabE      -----DGGDH PLLGMRMPHQ E----LVR-A HGKTSTTELL HPARGVLLDI ADDAEVREAA T------GWS   443
AlpK      -----GEGDH PLLGRRLPDR E----LVV-D GEKIPFYSLL RPGRAVLLEL GGDRGLRTAA A------GWA   438
PyrE3     ---------- ---GSRLPDV P----LVTAE GE-TSVYRLL EAGRGVLLDL GAGLPAVRHP ----------   415
PgaE      -----GTGSH PLLGKRMPAL E----LTT-A TRETSSTELL HTARGVLLDL ADNPRLRARA A------AWS   442
Grh06     -GPA-----H RLLGDFARDL R----LETEE G-PRTLPKFL RRGNFVLLDL AGRPEIAEEY AKWAAPLDWA   479
PHHY      LWMHFGDRLV TDGRFRIIVF AGKATDATQM SRIKKFAAYL DSE-NSVISR YTPKGADRNS RIDVITIHSC   565
MHBH      KPVQLGHCGK ADGRWRLYAF AAQNDLAQPE SGLLALCRFL EGDAASPLRR FTPAGQDIDS IFDLRAVFPQ   554
```

```
            477
RubL        ITARRVVPGT DAGAGTLADP DGDWSERYGG LRP------- ---------- -EGAVLVRPD GVVAWRS---   523
GrhO5       IAFHRVAP-- DAGEGRPVDV HGRWAEAY-G VGA------- ---------- -AGAVLVRPD GIVAWRS---   512
HlqO5       LAFHRIAP-- TAERGALADP DGRWADAY-G VGP------- ---------- -AGAVLVRPD GIVAWRV---   512
HyalO5      VTAWRVVAER ADEAGVLKDP EGLWADRY-G VGA------- ---------- -EGAVLVRPD GYVAWRS---   514
RdmE        VRAYRVGAG- ------LTDP ESAVSERY-G IGK------- ---------- -AGASLVRPD GIVAWRT---   512
PieE        VAVLPIGAG- ----GGPRDP YGTWAE-LRE VEE------- ---------- -SGAVLVRPD GHVAWRA---   567
HbpA        LKVCVIGPG- ----QEFVDT YGEYAK-ISE IGE------- ---------- -SGALLVRPD MFIAFRA---   568
RebC        TVTCH----- ---------- ----APEIHA LYE------- ---------- -RAHVLVRPD GHVAWRG---   529
RIFMO       DRVD------ HL-------- ----ADPGAA LD-------- ---------- -VPAALLRPD GHVAWV----   456
BexE        GRIDVVTARP AA-------- ----GRVPGA ---------- ---------- -TTAVLVRPD GHVAWAAP--   485
Tmn9        DRVDVVRAQP ---------- ------VPDL ---------- ---------- -PGTLLLRPD GCVAWHDGGG   476
OxyS        DRVDLVAAE- GV-------- ----ADPGSA VDG------- ---------- -LTALLVRPD GYICWT----   499
MtmOIV      DRVSVVAGE- AT-------- ----VEPPAQ ---------- ---------- ---ALLVRPD GYVAWA----   489
CabE        DRVDIVTASL HD-------- ----APPQGP LSD------- ---------- -ARAVLVRPD GYVAWISP--   478
AlpK        DRVDLVAAEF DG-------- ----CEAP-- ---------- ---------- -VDGILVRPD GYVAWVAALG   468
PyrE3       ---QVTYVRA ---------- ------EPTN RLD------- ---------- -ATAVLLRPD GVVAWRA---   443
PgaE        DRVDIVTAVP GE-------- ----VSATSG LRD------- ---------- -TTAVLIRPD GHVAWAAP--   477
GrhO6       GRVRHLVAT- CE-------- ----DEPELA ---------- ---------- ---GALIRPD GYVAWAADRD   508
PHHY        HRDDIEMHDF PAPALHPKWQ ---YDFIYAD CDSWHHPHPK SYQAWGVDET KGAVVVVRPD GYTSLVTDLE   627
MHBH        AYTEVALETL PALLLPPKGQ LGMIDYEKVF SPDLKNAGQD IFELRGIDRQ QGALVVVRPD QYVAQVLPLG   619
```

```
                  526
RubL        -------PGA DPGGEES--A VLAAVLRSVL ARESRTGGK- ------   555
GrhO5       -------RDG MPGGAGG--R ALTAALRTVL AR-------- ------   537
HlqO5       -------TGA VPSGEQG--A VLEAALRRIL AR-------- ------   537
HyalO5      -------DGA EPAGDET--G VLTEVLRAVL ARI------- ------   540
RdmE        -------DEA AADAAQTLEG VLRRVLDR-- ---------- ------   535
PieE        -------RD- -HGHAKELPE VMARVLHQPD PAARRTGGPG A-----   601
HbpA        -------KD- --ASREGLEQ LNVAVKSILG RA-------- ------   592
RebC        -------DHL PAELGGLVDK VRGAA----- ---------- ------   549
RIFMO       GEDQDDLLAH LPRWFGAAT- ---------- ---------- ------   476
BexE        GTHA----DL PMALERWFGP APR------- ---------- ------   507
Tmn9        WGQ----DEL RTALRTWFGA PTG------- ---------- ------   500
OxyS        AAPETGTDGL TDALRTWFGP PAM------- ---------- ------   523
MtmOIV      GSPAATADEL RASLARWFGP PANREPVGHQ ERAGRRGRPL SALKPE   536
CabE        GSRA----GL TEALDRWFGP AR-------- ---------- ------   499
AlpK        AGAD----GL TTALDRWFGP TA-------- ---------- ------   491
PyrE3       -PQ----DGL EAALETWFGP AA-------- ---------- ------   462
PgaE        GSHH----DL PMALERWFGA PLTG------ ---------- ------   500
GrhO6       ASPAEIAEGL RTAIETWAGI TDPTRAVFS- ---------- ------   542
PHHY        GTAEIDRYFS GILVEPKEKS GAQTEADWTK STA------- ------   665
MHBH        DHAALSAYFE SFMRA----- ---------- ---------- ------   639
```

Figure S1: Uncropped structure-based multiple sequence alignment (cf. Figure 3 main text) showing the spiroketal synthases RubL and GrhO5, as well as previously crystallized group A monooxygenases. In addition, GrhO5/RubL homologs from heliquinomycin (HlqO5) and hyaluromycin (HyalO5) biosynthesis and the second group A monooxygenase involved in griseorhodin A biosynthesis (GrhO6) were included. The structure-based sequence alignment was generated using the online tool T-coffee[4] and visualized in SeaView[6]. Final color editing to highlight important amino acid residues (FAD-binding and solvent gate keeping) and structurally conserved regions (*blue* boxes) was carried out in PowerPoint. For PDB-IDs of the proteins included in the alignment, see Table S2.

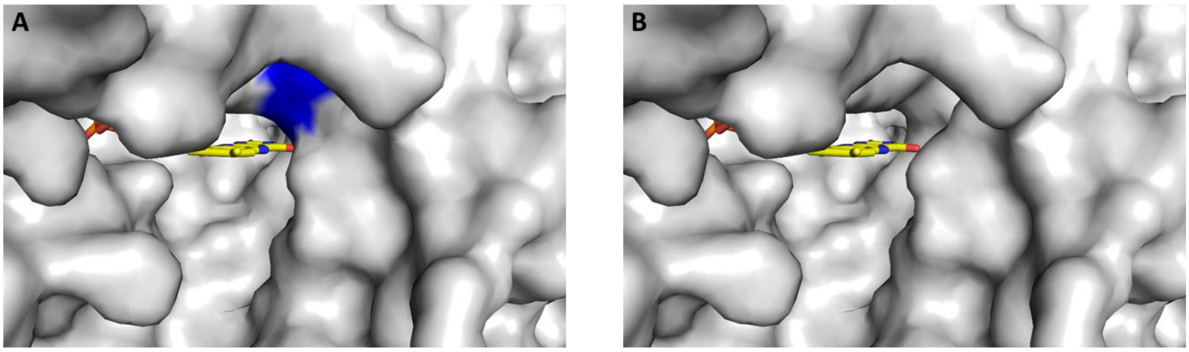

Figure S2: Surface representation of PHHY indicating the putative gate-keeping role of Trp336. While Trp336 (indicated in *blue*) shields the active site in the wild type enzyme (**A**), the replacement of the same residue by an Ala would lead to a strongly increased solvent accessibility of the cofactor-binding cleft (**B**).

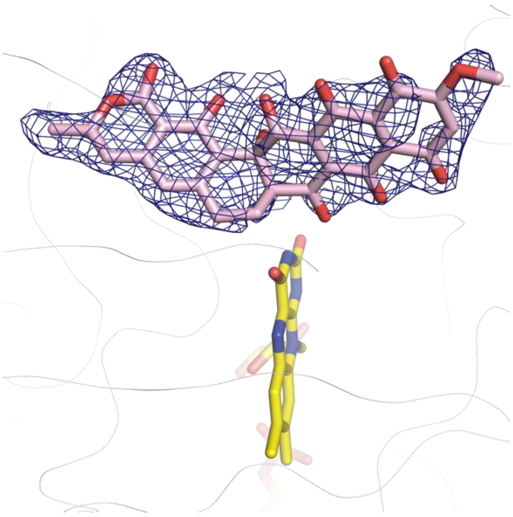

Figure S3: GrhO5 ternary complex structure with FAD and collinone (3). Polder omit electron density maps (*blue mesh*) for collinone (**3**) was contoured at 3 σ above the mean (collinone was positioned as stick model in the extra densities, but not included in the refinement).

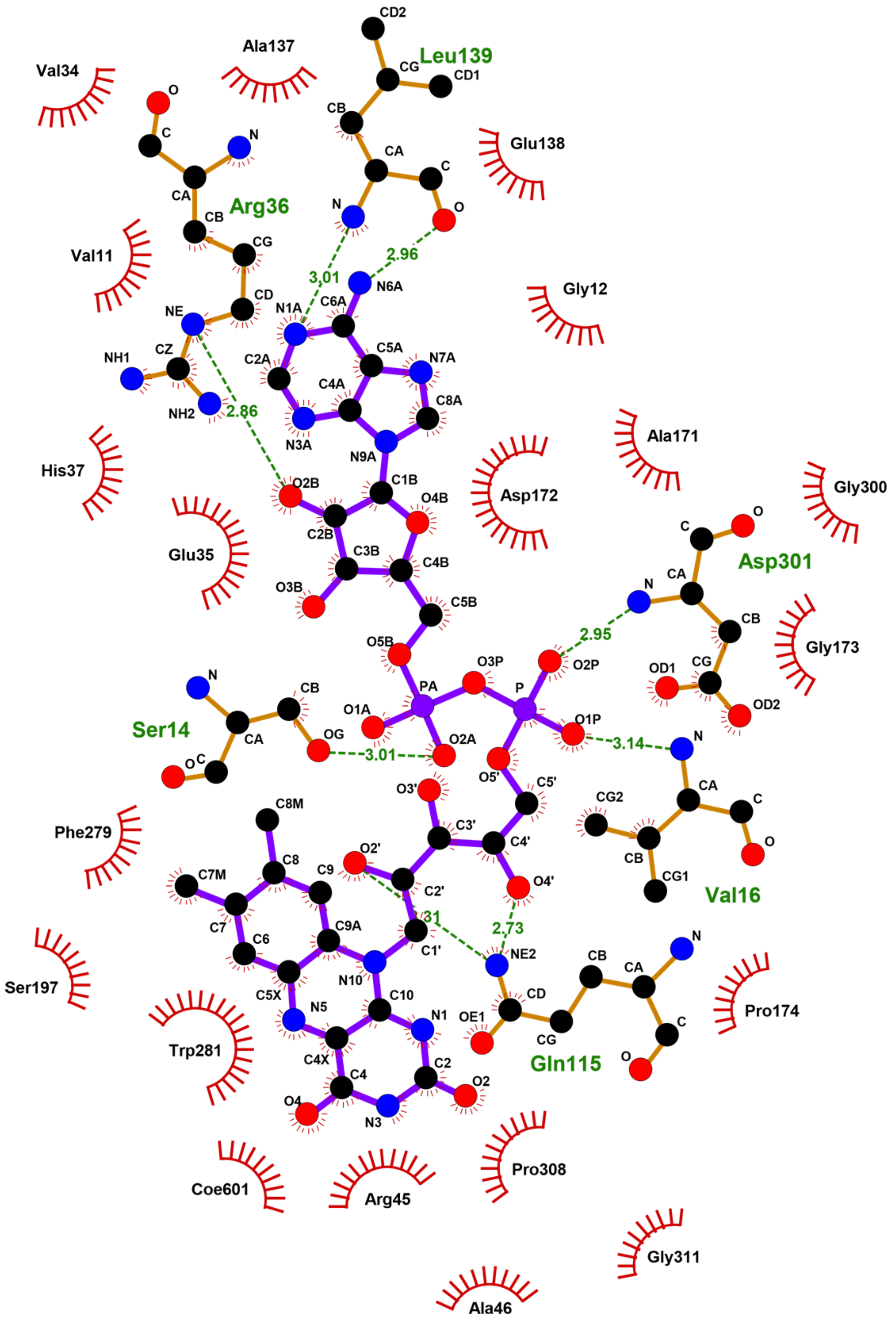

Figure S4: LigPlot showing the interactions of GrhO5 (3-soaked)-bound FAD ("OUT"-conformation) with the amino acid backbones and side-chains in the active site.

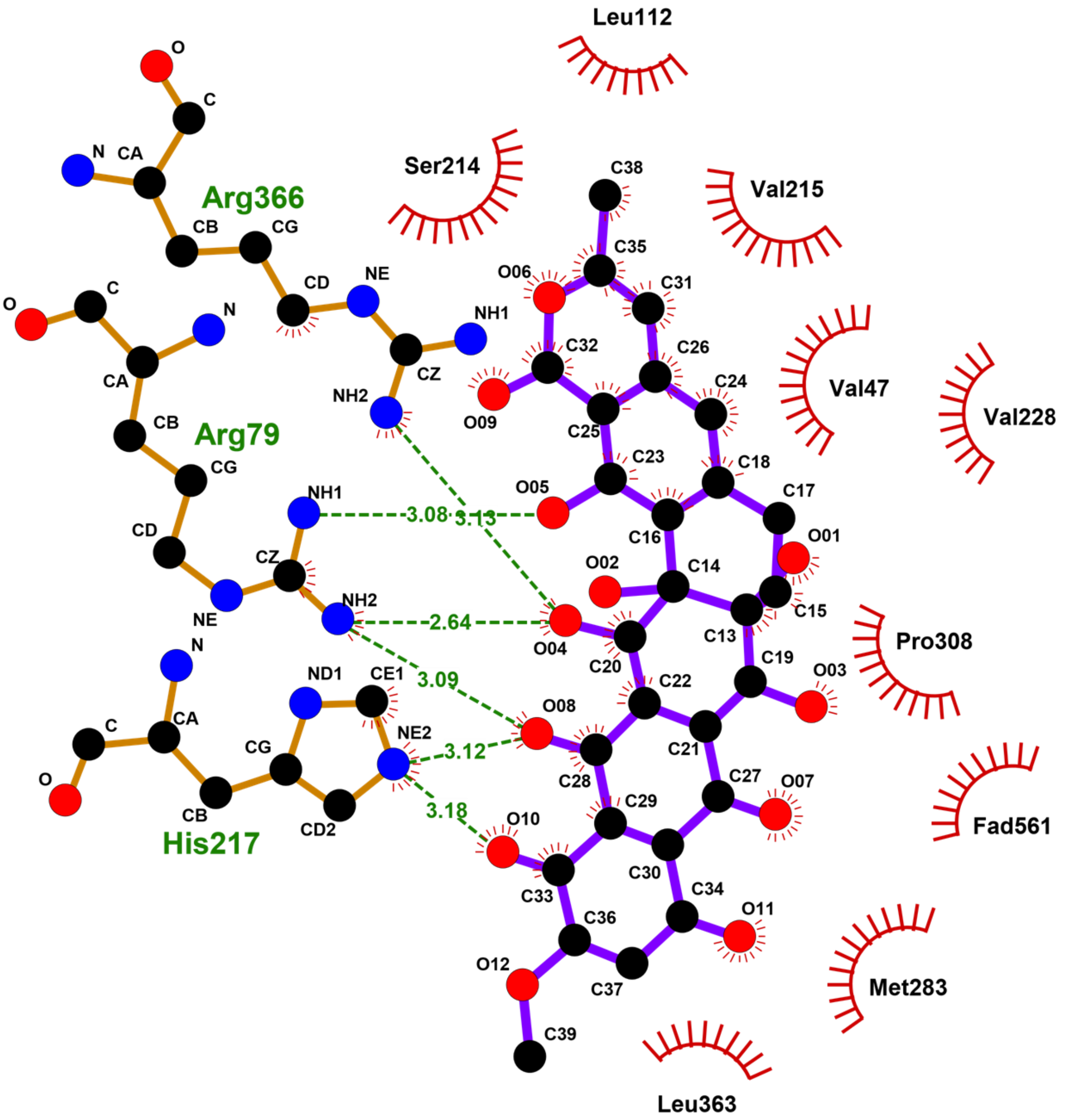

Figure S5: LigPlot showing the interactions of GrhO5-bound collinone (3) with the active site residues Arg79, His217 and Arg366. See main text for details.

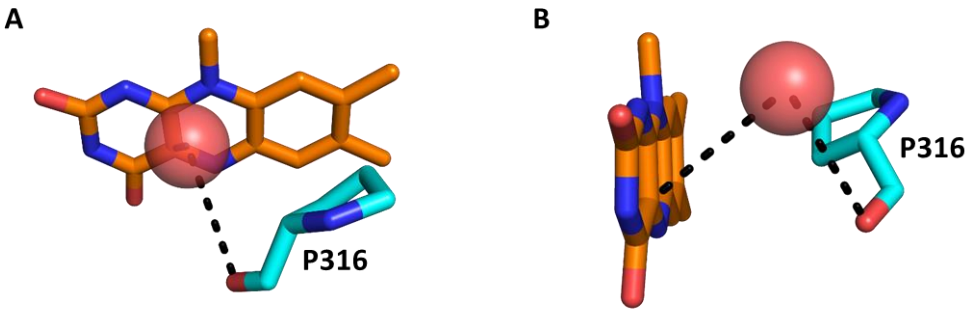

Figure S6: Putative O2-reaction site in RubL shown in face-on (A) and side-view (B). The Cl^--ion observed in the crystal structure of RubL may well indicate the superoxide binding site in the spiroketal synthase as it is found in about 4 Å distance to the C4a-position of the FAD cofactor, which is within the optimal range for covalent oxygen adduct formation between the superoxide and the flavin (in its semiquinonic form).

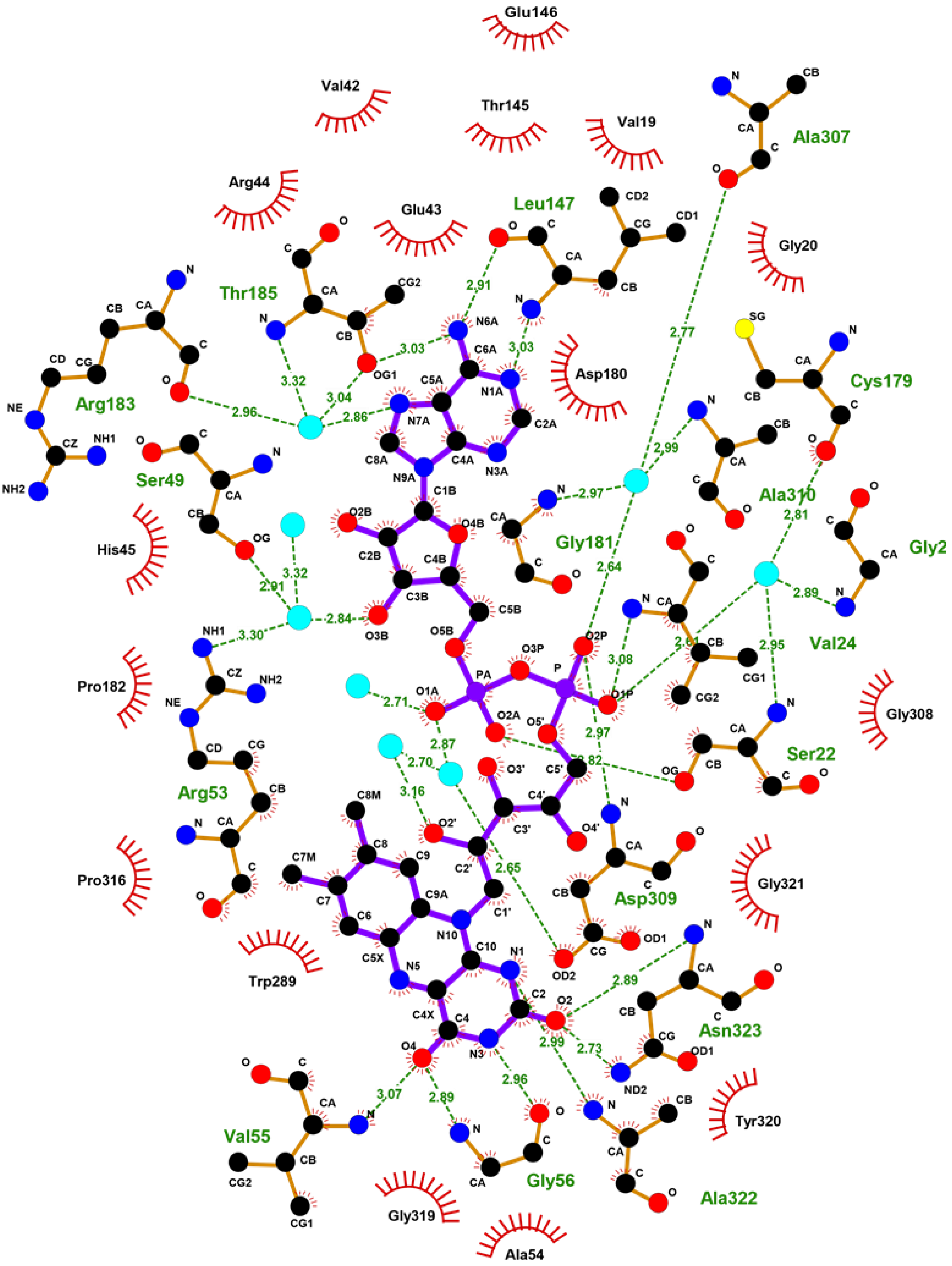

Figure S7: LigPlot showing the interactions of RubL-bound FAD ("IN"-conformation) with the amino acid backbones and side-chains in the active site. The backbones of V55 and G56 as well as of N323, A322 and G319 (not visible from the LigPlot) interact with the C4=O and C2=O of FAD, respectively. Similarly, interactions of the backbones of A322 as well as of G56 and G319 (not visible from the LigPlot) with the N1 and N3, respectively, are observed.

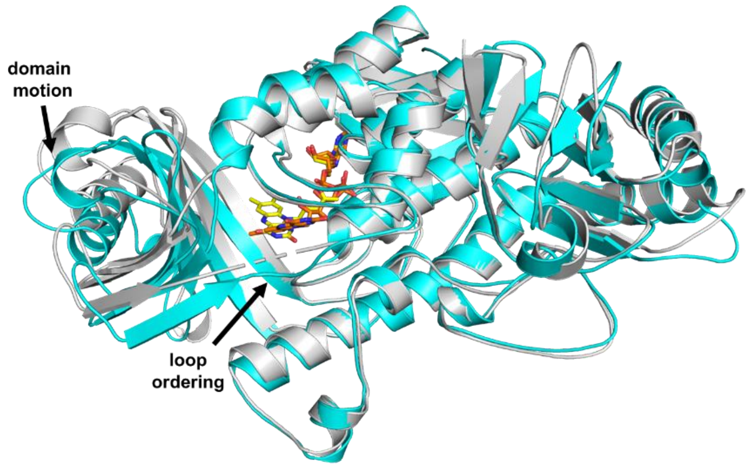

Figure S8: Overlay of the overall structures of GrhO5 (grey) and RubL (teal) with the FAD-cofactor in "OUT"- (yellow) and "IN"- (orange) conformation, respectively. A, The overlay of the overall structures of GrhO5 and RubL indicates that movement of the FAD-cofactor from the "OUT" to the "IN" position goes along with a shift of domain II and the ordering of the loop region between residues 90-110 when co-crystallized with substrate analog **7**.

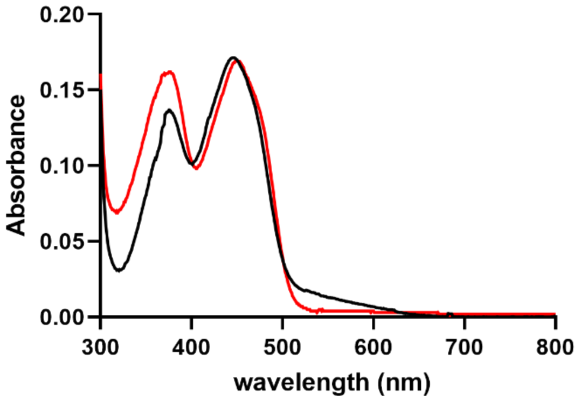

Figure S9: Comparison of the spectral properties of RubL wild type and the W289A variant. While the UV-visible absorption spectrum of RubL wild type (*black line*), apart from the characteristic flavin absorption maxima at 370 and 450 nm, exhibits a long-wavelength absorption between 500 and 650 nm, the latter is no longer observed in the W289A variant (*red line*). Therefore, W289 very likely determines the spectral characteristics of the wild-type enzyme, which is in line with the observed sandwiched π-π interaction between the isoalloxazine ring and the indole moiety of W289 in the crystal structure.

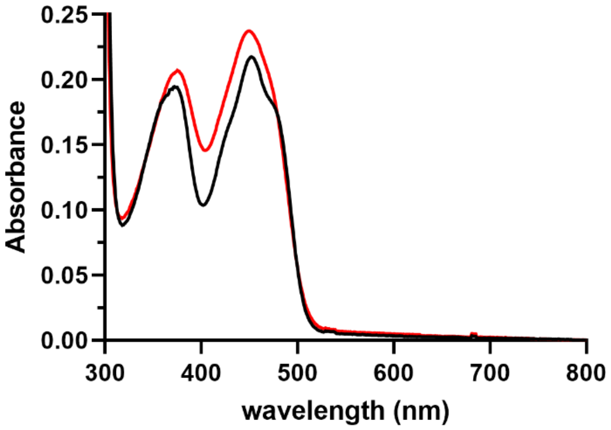

Figure S10: UV-visible absorption spectra of native (black) and denatured (red) GrhO6. The spectrum of native GrhO6 was recorded between 300 and 800 nm in 50 mM Tris, 300 mM NaCl, 10 % glycerol, pH 7.4 prior to the addition of 0.5 % SDS to release the FAD-cofactor for determination of the extinction coefficient of GrhO6-bound FAD.

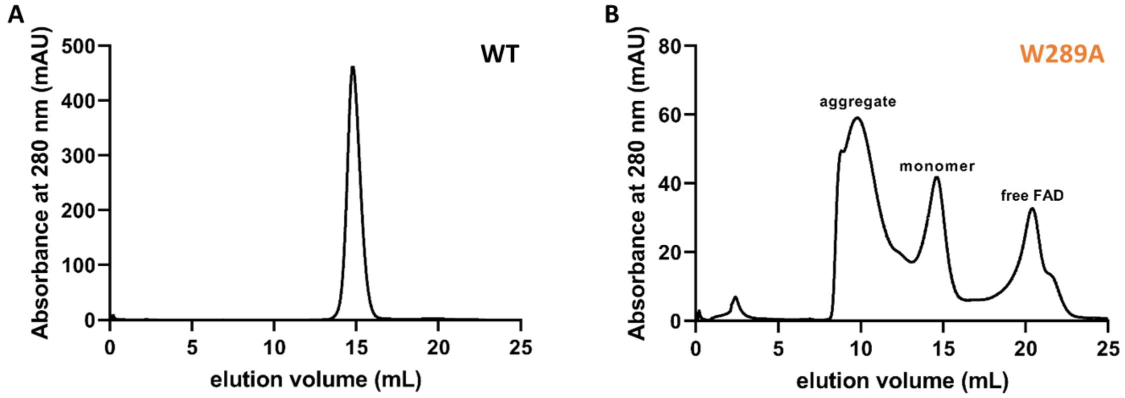

Figure S11: FPLC-chromatograms recorded in the course of the gel-filtration of RubL wild type (A) and the W289A-variant (B). While only a clear peak corresponding to monomeric enzyme (~15 mL; ~60 kDa) is observed in the gel-filtration chromatogram of the RubL wild type, additionally, both a substantial aggregate peak at ~10 mL and a peak corresponding to free FAD (~20 mL) are found in the elution profile of RubL-W289A.

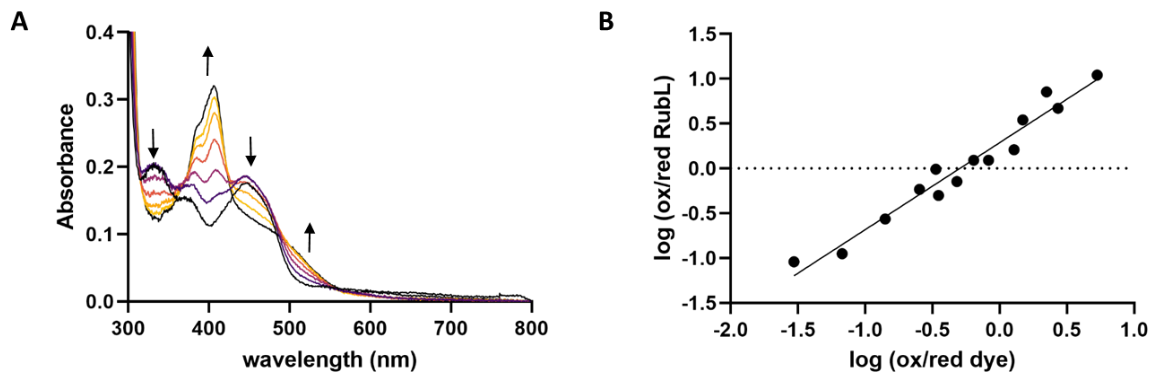

Figure S12: Determination of the reduction potential of RubL wild type. A, Selected UV-visible absorption spectra recorded in the course of the xanthine oxidase catalyzed reduction of the RubL-bound FAD cofactor and the reporting dye anthraquinone-2,6-disulfonate (E°: -184 mV;[23]). **B**, Nernst-plot generated by plotting the log(ox/red) of the protein-bound FAD (at 357 nm) as a function of the log(ox/red) of the dye (at 343 nm). Using the value of the y-axis intercept, subsequently the midpoint potential of RubL was calculated (-177 ± 1 mV; from 4 determinations). Due to the overlapping spectra, the reduction potential was calculated based on the spectral changes at the isobestic points observed in the course of the separate reduction of RubL (343 nm) and anthraquinone-2,6-disulfonate (357 nm).

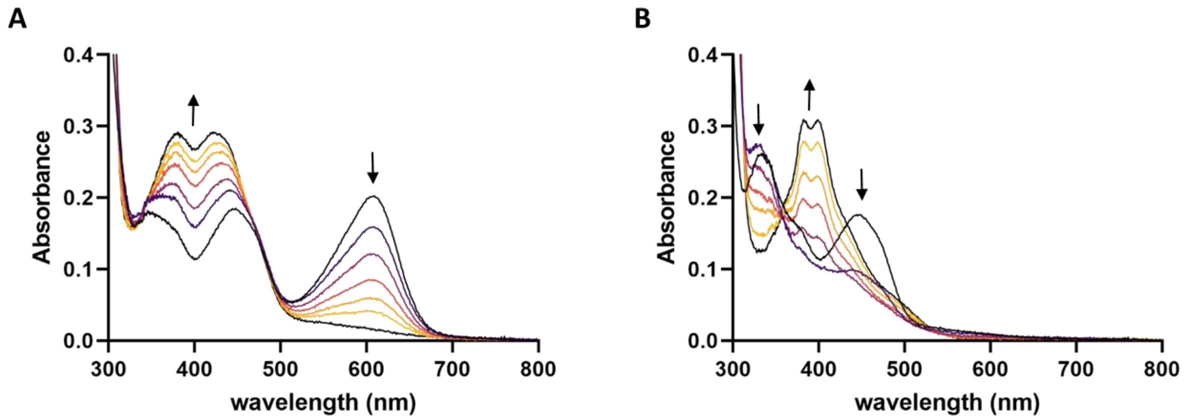

Figure S13: Estimation of the upper and lower boundary of the reduction potential of RubL-bound FAD. A, Selected UV-visible absorption spectra recorded in the course of the xanthine oxidase catalyzed reduction of the RubL-bound FAD cofactor and the reporting dyes indigo carmine (**A**; E°, -125 mV[24]) and anthraquinone-2-sulfonate (**B**; E°, -225 mV[25]). **A**, The spectral changes indicate that only the reporting dye, but not the RubL-bound FAD became reduced, suggesting a more negative reduction potential (than -125 mV) for the enzyme-bound cofactor. **B**, The spectral changes indicate that first only the protein-bound cofactor and later the reporting dye became reduced, suggesting a more positive reduction potential (than -225 mV) for the enzyme-bound cofactor.

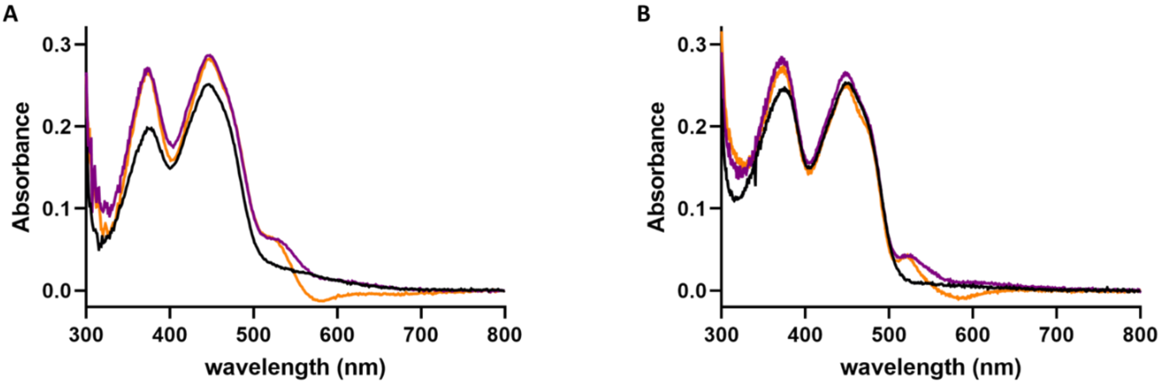

Figure S14: UV-visible absorption spectra of RubL WT (A) and RubL-W289A (B) in the absence and presence of 5 and NADP+. The *black* spectrum corresponds to native RubL-WT (A) and RubL-W289A (B) in 50 mM Tris, 300 mM NaCl, pH 7.4, while the *purple* spectra were recorded in the presence of **5** (free **5** was used as blank). Upon **5** addition, the original charge-transfer interaction (between FAD and W289) remains intact for the RubL-WT while an additional charge-transfer interaction is established. The further addition of 1 mM $NADP^+$ to the enzyme-**5** mixture (*orange* lines) resulted in a significant loss of long-wavelength absorption (particularly for the WT-enzyme), suggesting that $NADP^+$ binding affects the interaction of W289 with the FAD cofactor.

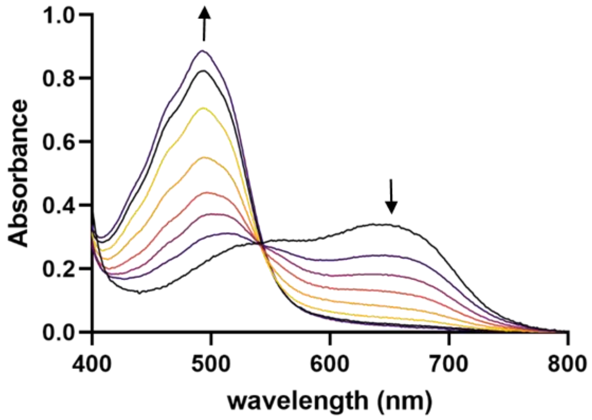

Figure S15: Selected (UV-)visible absorption spectra recorded in the course of collinone (3) reduction. Upon reduction of **3** in buffer pH 7.4 (under anoxic conditions), a rapid decrease of the absorption between 550-800 nm and an strong increase of the absorption between 400 and 550 nm are observed.

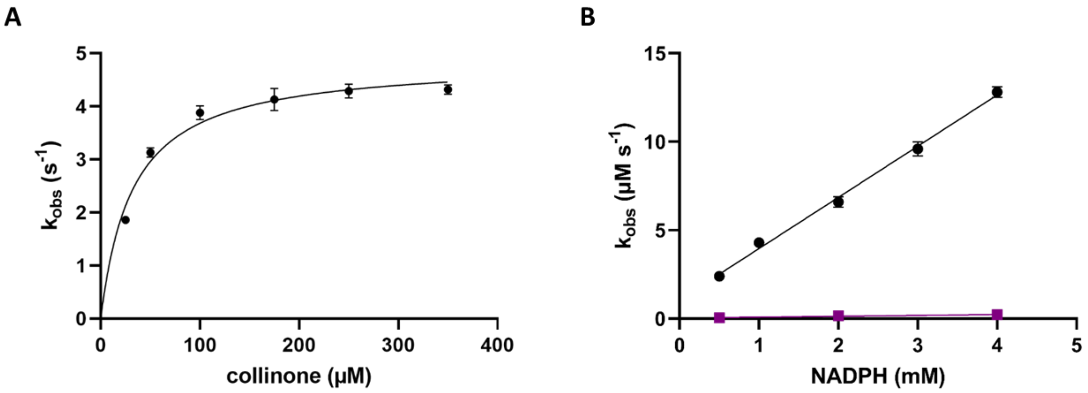

Figure S16: Basic kinetic characterization of the first step of collinone (3) turnover (reduction to dihydrocollinone (5)). A, Michaelis-Menten plot generated from the initial rates determined at six different **3** concentrations between 25 and 350 µM (rates at each substrate concentration were determined at least in triplicate; standard deviations are shown as error bars). By applying a hyperbolic fit, the enzyme typical kinetic parameters k_{catapp} (4.6 ± 0.2 s^{-1}) and K_{Mapp} (22 ± 4 s^{-1}) were obtained. **B**, Effect of the NADPH-concentration on **3** reduction. Interestingly, the rate of **5** formation linearly increased with the NADPH concentration (0.5 - 4 mM), both in the absence (*purple* squares) and in the presence (*black* spheres) of 1 µM RubL, indicating weak or only transient binding of NADPH to the RubL-**3** complex (the rates at each NADPH-concentration were determined at least in triplicate; standard deviations are displayed as error bars).

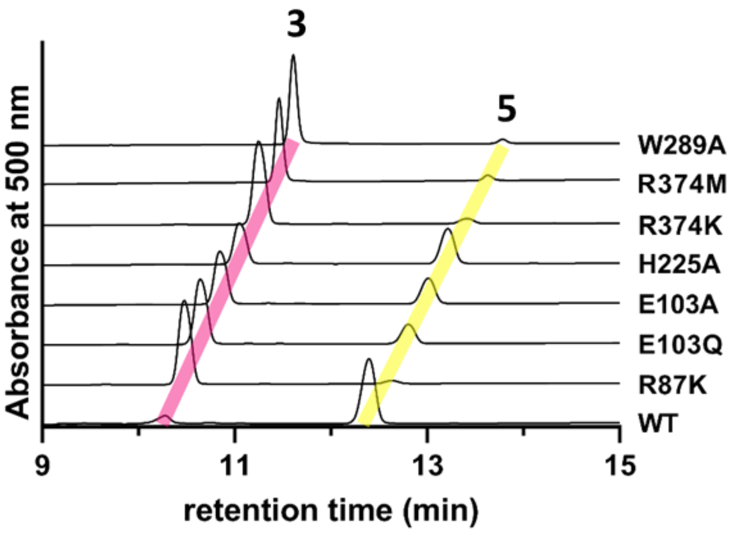

Figure S17: HPLC-analysis of the conversion of 3 (10.3 min, pink line) into 5 (12.4 min, yellow line) catalyzed by RubL wild type or several active site variants. To investigate the influence of several amino acid replacements on the rate of **3** reduction, RubL wild type and seven variants (each 10 µM) were separately mixed with a 350 µM **3** solution (in the presence of 1.5 mM NADPH) and incubated at 30°C and 750 rpm for 30 s under aerobic conditions. Then, the reactions were stopped with EtOAc:FA (9:1) and the organic layers immediately analyzed by HPLC-DAD. While almost full conversion of **3** into **5** was observed for the wild type enzyme, particularly the arginine-variants (R87K, R374K, and R374M) and the tryptophan-variant (W289A) showed a strongly reduced reduction activity. Note that in the shown WT trace, **3** was almost completely converted to **5** without detectable amounts of the final products **4** and **7** with retention times of 14.6 min and 10.1 min, respectively (compare Figure S18). This suggests that enzyme-bound **5** is most likely displaced by remaining **3** before hydroxylation can occur.

Table S3: Summary of the relative collinone (3) reduction rates determined under anoxic (column 2 and 3) and aerobic conditions (column 4). Both in the photometric, as well as in the HPLC-based assays, very similar effects of the amino acid replacements on **3** reduction were observed. While the substitution of E103 or H225 resulted in residual activities of 20-30 %, the replacement of R87, R374 or W289 diminished the reduction activity to 3-10 %. Interestingly though, for some of the variants the relative reduction rates at the lower **3** concentration of 50 µM were slightly higher rather than lower, despite the anticipated role of both arginine residues in substrate binding (however, evaluation of the data at a substrate concentration of 50 µM is challenging, as the absorption at 650 nm (λ_{max}) is close to the detection limit).

**Protein**	**relative activity (%)** 50 µM **3**	**relative activity (%)** 350 µM **3**	**HPLC-assay collinone$_{red}$ (%)**
**WT**	100 ± 3	100 ± 2	85 ± 2
**R87K**	7 ± 0	3 ± 0	4 ± 0
**E103Q**	19 ± 1	22 ± 3	21 ± 2
**E103A**	25 ± 1	26 ± 2	25 ± 1
**H225A**	37 ± 1	26 ± 1	32 ± 2
**R374K**	10 ± 1	7 ± 0	7 ± 1
**R374M**	3 ± 0	3 ± 0	7 ± 1
**W289A**	6 ± 0	5 ± 0	5 ± 1

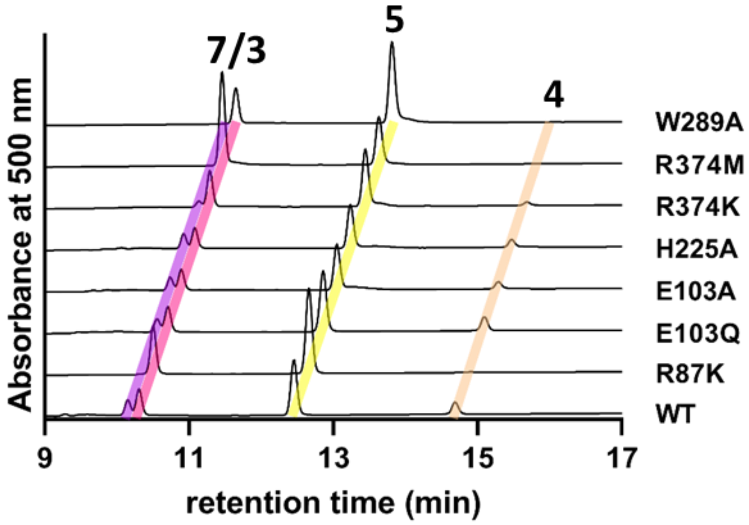

Figure S18: HPLC-analysis of the conversion of 3 (10.3 min, pink line) via 5 (12.4 min, yellow line) into 4 (14.6 min, light orange line) and 7 (10.1 min, light blue line) catalyzed by RubL wild type or several active site variants. To investigate the influence of several amino acid replacements on the efficiency of **3** turnover and **4**/**7** formation, RubL wild type and seven variants (10 µM) were separately mixed with a 350 µM **3** solution (in the presence of 1.5 mM NADPH) and incubated at 30°C and 750 rpm for 10 min. Then, the reactions were stopped with EtOAc:FA (9:1) and the organic layers immediately analyzed by HPLC-DAD. While substantial conversion of **3** into **4** and **7** was observed in assays with the wild type protein as well as the glutamine and the histidine variants, turnover was significantly lower in the reactions containing the R374K-variant. No reaction products were found in the assay mixtures containing the W289A-, the R87K- or the R374M variant, suggesting a crucial role of these residues in catalysis.

Table S4: Summary of the 3 turnover-efficiency observed for RubL wild type and several active site variants. Reactions were quenched before full turnover occurred to allow for comparison of the reaction rates. Shown are relative amounts of residual **3** in the assay mixture (column 1) and of produced compounds **5**, **7** and **4** (columns 2, 3 and 4, respectively). n.d. = not detectable.

	substrate **3**	intermediate **5**	shunt product **7**	product **4**
**WT**	23 ± 0	53 ± 1	13 ± 1	12 ± 1
**R87K**	36 ± 3	61 ± 2	n.d.	< 0.5
**E103Q**	23 ± 1	56 ± 1	10 ± 1	12 ± 0
**E103A**	20 ± 1	57 ± 2	11 ± 1	14 ± 2
**H225A**	20 ± 1	56 ± 2	13 ± 1	11 ± 1
**R374K**	31 ± 3	59 ± 1	6 ± 1	4 ± 1
**R374M**	62 ± 2	38 ± 2	n.d.	n.d.
**W289A**	23 ± 3	77 ± 2	n.d.	n.d.

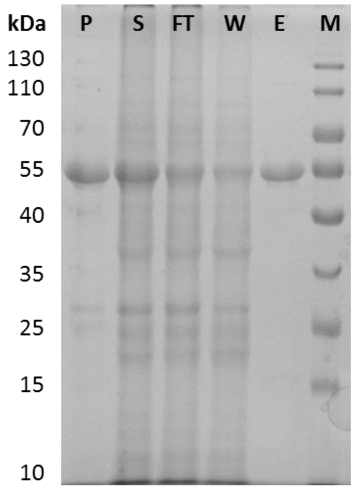

Figure S19: SDS-PAGE analysis of the different fractions collected in the course of the purification of a selected RubL-variant. Shown are: lane 1, cell pellet after lysis (P); lane 2, cleared lysate (S); lane 3, column flow-through (FT); lane 4, wash fraction (W); lane 5, selected elution fraction; lane 6, prestained protein ladder (M, Thermo Fisher Scientific®).

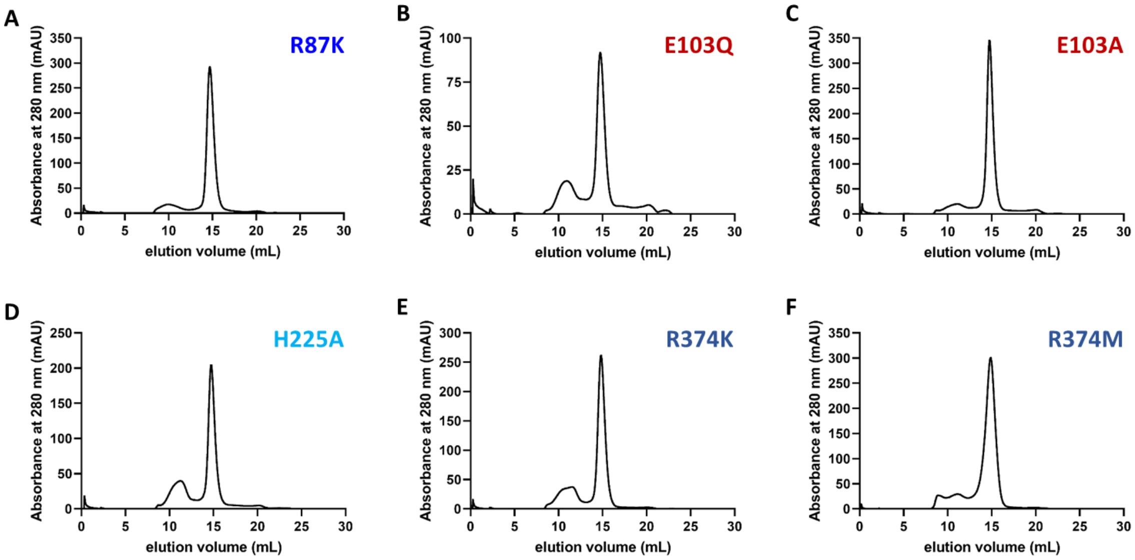

Figure S20: Analytical size exclusion chromatography of different RubL-variants. Shown are the chromatograms for all analyzed RubL-variants (**A**, R87K; **B**, E103Q; **C**, E103A; **D**, H225A; **E**, R374K; **F**, R374M) using a Superdex200 GL10/300 column pre-equilibrated with 20 mM Tris, 50 mM NaCl, pH 7.4. The sharp peak at 15 mL (~60 kDa) indicates that the major fraction of all variants is present as monomeric protein in solution (like the wild type) and that the single amino acid replacements introduced in the active site of RubL did not have any adverse effects on protein folding. For wild type enzyme and the W289A variant, see Figure S12.

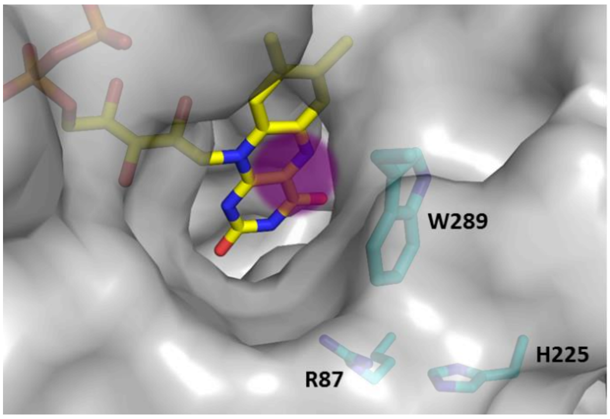

Figure S21: Surface representation of RubL, showing the NADPH-entry to the active site and the FAD-cofactor modeled in OUT-position. The purple sphere indicates the putative NADPH-reaction site.

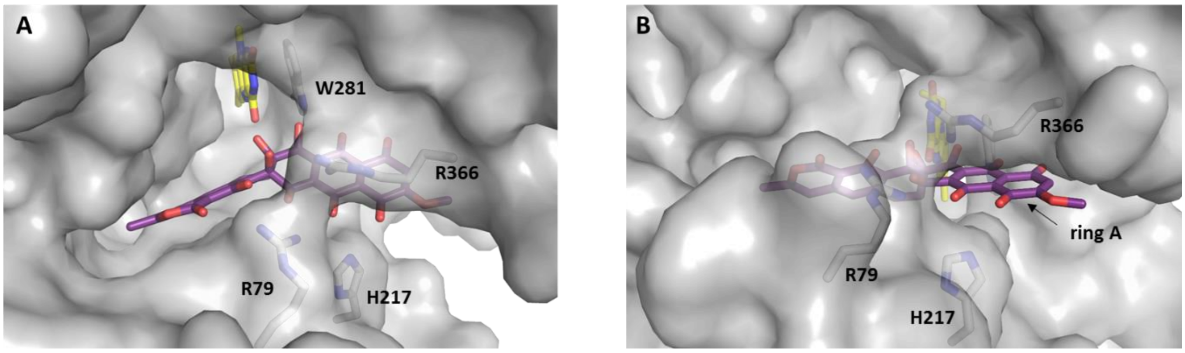

Figure S22: Surface representation of 3-soaked GrhO5 with the FAD-cofactor (yellow), 3 (purple) and the amino acid side chains coordinating 3/5 (grey) shown as sticks. Both views demonstrate the rather open active site of GrhO5, even in the presence of **3/5** and indicate that ring A of **3** can easily be accessed by NADPH, allowing for direct hydride transfer and formation of **5** independent of the FAD cofactor.

3. References

[1] B. Frensch, T. Lechtenberg, M. Kather, Z. Yunt, M. Betschart, B. Kammerer, S. Lüdeke, M. Müller, J. Piel, R. Teufel, *Nat. Commun.* **2021**, *12*, 1431.

[2] in *Flavins and Flavoproteins 1990*. *Proceedings of the Tenth International Symposium, Como, Italy, July 15-20, 1990* (Eds.: B. Curti, S. Ronchi, G. Zanetti), De Gruyter, Berlin, Boston, **1991**, pp. 59–66.

[3] K. Minnaert, *Biochimica et biophysica acta* **1965**, *110*.

[4] F. Armougom, S. Moretti, O. Poirot, S. Audic, P. Dumas, B. Schaeli, V. Keduas, C. Notredame, *Nucleic Acids Res.* **2006**, *34*, W604-8.

[5] A. M. Waterhouse, J. B. Procter, D. M. A. Martin, M. Clamp, G. J. Barton, *Bioinformatics (Oxford, England)* **2009**, *25*, 1189.

[6] M. Gouy, S. Guindon, O. Gascuel, *Mol. Biol. Evol.* **2010**, *27*, 221.

[7] I. Letunic, P. Bork, *Nucleic Acids Res.* **2019**, *47*, W256-W259.

[8] W. Kabsch, *Acta Crystallogr. D Biol. Crystallogr.* **2010**, *66*, 125.

[9] P. Evans, *Acta Crystallogr. D Biol. Crystallogr.* **2006**, *62*, 72.

[10] A. J. McCoy, R. W. Grosse-Kunstleve, P. D. Adams, M. D. Winn, L. C. Storoni, R. J. Read, *J. Appl. Crystallogr.* **2007**, *40*, 658.

[11] L. Jaroszewski, Z. Li, X.-h. Cai, C. Weber, A. Godzik, *Nucleic Acids Res.* **2011**, *39*, W38-44.

[12] A. Waterhouse, M. Bertoni, S. Bienert, G. Studer, G. Tauriello, R. Gumienny, F. T. Heer, T. A. P. de Beer, C. Rempfer, L. Bordoli et al., *Nucleic Acids Res.* **2018**, *46*, W296-W303.

[13] Y. Lindqvist, H. Koskiniemi, A. Jansson, T. Sandalova, R. Schnell, Z. Liu, P. Mäntsälä, J. Niemi, G. Schneider, *J. Mol. Biol.* **2009**, *393*, 966.

[14] P. Emsley, K. Cowtan, *Acta Crystallogr. D Biol. Crystallogr.* **2004**, *60*, 2126.

[15] G. N. Murshudov, P. Skubák, A. A. Lebedev, N. S. Pannu, R. A. Steiner, R. A. Nicholls, M. D. Winn, F. Long, A. A. Vagin, *Acta Crystallogr. D Biol. Crystallogr.* **2011**, *67*, 355.

[16] P. D. Adams, P. V. Afonine, G. Bunkóczi, V. B. Chen, I. W. Davis, N. Echols, J. J. Headd, L.-W. Hung, G. J. Kapral, R. W. Grosse-Kunstleve et al., *Acta Crystallogr. D Biol. Crystallogr.* **2010**, *66*, 213.

[17] D. Liebschner, P. V. Afonine, N. W. Moriarty, B. K. Poon, O. V. Sobolev, T. C. Terwilliger, P. D. Adams, *Acta Crystallogr. D Struct. Biol.* **2017**, *73*, 148.

[18] N. W. Moriarty, R. W. Grosse-Kunstleve, P. D. Adams, *Acta Crystallogr. D Biol. Crystallogr.* **2009**, *65*, 1074.

[19] C. Enroth, H. Neujahr, G. Schneider, Y. Lindqvist, *Structure* **1998**, *6*, 605.

[20] T. Hiromoto, S. Fujiwara, K. Hosokawa, H. Yamaguchi, *J. Mol. Biol.* **2006**, *364*, 878.

[21] M. Kanteev, A. Bregman-Cohen, B. Deri, A. Shahar, N. Adir, A. Fishman, *Biochim. Biophys. Acta* **2015**, *1854*, 1906.

[22] A. H. Westphal, D. Tischler, W. J. H. van Berkel, *Arch. Biochem. Biophys.* **2021**, *702*, 108820.

[23] A. E. Remick, *J. Chem. Educ.* **1961**, *38*, 158.

[24] P. G. Tratnyek, T. E. Reilkoff, A. W. Lemon, M. M. Scherer, B. A. Balko, L. M. Feik, B. D. Henegar, *Chem. Educator* **2001**, *6*, 172.

[25] P.-L. Hagedoorn, L. van der Weel, W. R. Hagen, *J. Vis. Exp.* **2014**, e51611.
